# 44-year journey (1980–2024): scientometric insights into Sigesbeckiae herba and update on its medicinal properties and phytochemicals profile

**DOI:** 10.1186/s13020-025-01308-6

**Published:** 2026-03-04

**Authors:** Parsa Dar, Cui Wenqing, Shaden A. M. Khalifa, Junming Chen, Kegang Linghu, Wei Xiong, Hesham R. El-Seedi, Hua Yu

**Affiliations:** 1 Institute of Chinese Medical Sciences, State Key Laboratory of Mechanism and Quality of Chinese Medicine, University of Macau, Macao, China; 2 Neurology and Psychiatry and Department, Capio Saint Göran’s Hospital, Sankt Göransplan 1, 112 19 Stockholm, Stockholm, Sweden; 3 School of Food and Biological Engineering, Jiangsu University, Zhenjiang, 212013 China; 4 The High Efficacy Application of Natural Medicinal Resources Engineering Center of Guizhou Province & School of Pharmaceutical Sciences, Guizhou Medical University, Guian New District, Guiyang, 561113 Guizhou China; 5 School of Pharmacy, Shenzhen University Medical School, Shenzhen University, Shenzhen, 518055 China; 6 Department of Chemistry, Faculty of Science, Islamic University of Madinah, 42351 Madinah, Saudi Arabia; 7 Macao Centre for Research and Development in Chinese Medicine, Institute of Chinese Medical Sciences, University of Macau, Macao, SAR China

**Keywords:** Scientometric analysis, Sigesbeckiae Herba, *Sigesbeckia orientalis* L., *S. glabrescens* Makino, *S. pubescens* Makino, Anti-inflammatory, *Ent*-pimrane, Rheumatic arthritis

## Abstract

This study uses Scientometric analysis to review Sigesbeckiae Herba (SH) research worldwide from 1980 to 2024. Data-driven analysis identifies patterns, partnerships, and possibilities. Due to its wide profile and traditional medical uses, SH is gaining attention, according to this research. China has made the most important contributions to SH research, followed by the United stated of America and Korea. Countries and organizations have not collaborated on SH research. We found “anti-inflammation,” “rheumatic arthritis,” and “NF-κβ” using keyword co-occurrence analysis. Citation analysis showed SH research's most prominent institutions, journals, and authors, offering a complete snapshot of organization and diffusion. The data shows that this subject has received substantial academic interest despite a reduction in publications since 2019. These findings highlight the need for cooperation, multidisciplinary methods, and attention to underrepresented disciplines for academics, funding organizations, and policymakers. SH has been used to cure inflammation. *Sigesbeckia orientalis* L., *S. pubescens* Makino, and *S. glabrescens* Makino are the primary botanical sources. This research extensively evaluates SH literature, focusing on its botanical properties, bioactive components, pharmacological effects, and probable toxicity. More than three hundred phytochemicals, including flavonoids, diterpenoids, sesquiterpenoids, oxylipins, and lignoids, were identified in the extract, many of which are known to exert diverse biological effects. The extracts of SH are used in traditional Chinese medicine (TCM) due to its immunomodulatory, anti-inflammatory, and anti-cancer activities and are being considered for cosmetics and skin issues. Pharmacological benefits of diterpenoids, sesquiterpenoids, and flavonoids are found. The study recommends further research and cooperation to enhance TCM and herbal research by comparing the chemical and pharmacological properties of these three SH species.

**Supplementary Information:**

The online version contains supplementary material available at 10.1186/s13020-025-01308-6.

## Introduction

Sigesbeckiae Herba (SH), a member of the Asteraceae family comprising 1000 genera and 25,000–30,000 species, is referred to as ‘Xi-Xian Cao’ or St. Paul’s wort. It has been integral to traditional Chinese medicine since the Tang dynasty, as recorded in the ancient medical text ‘‘*Newly Revised Materia Medica’’* and later in works such as Classified Materia Medica from Historical Classics for Emergency [1–3]. Despite this recognition, the three species differ significantly in their phytochemical profiles and pharmacological activities, which has implications for their therapeutic applications. Sigesbeckia is named for a botanist Johann Georg Sigesbeck, who was a strong critic of Carl Linnaeus 's botanic classification system. He was known to refer to it as "loathsome harlotry" because of the focus of the system upon the presence of (or lack of) sex organs in plants and their locations and groupings [1, 2] In 1963, *Sigesbeckia pubescens* Makino (SP) was initially recognised in the Pharmacopeia of China and endorsed as SH; subsequently, in 1977, *S. orientalis* L. (SO) and *S. glabrescens* Makino (SG) were also documented and suggested as SH [1]. SH has been pharmacologically established to produce a diverse array of biological effects, which include antioxidant and anti-inflammatory activities, anti-allergic properties, anti-thrombotic and anti-atherosclerotic actions, antimicrobial effects, enhancement of microcirculation, stimulation of skin wound healing, and anti-cancer potential [4–16]. It is a therapeutically utilized traditional Chinese medicine with remarkable therapeutic effectiveness. Notably, kirenol is a shared bioactive compound found in all three species, while others such as darutoside (SP), germacranolides (SO), and glabrescolide A (SG) exhibit species-specific distribution. These differences underscore the need for a consolidated comparative table in the Phytochemistry and Pharmacology sections. SH is a traditional Chinese medicine known for its strong therapeutic effects and clinical effectiveness. SH can alleviate arthritis via multiple mechanisms due to its vast array of active constituents [17]. The extract of SO is chiefly recognized for its skin conditioning qualities. It effectively enhances the skin's appearance and texture by ensuring it is adequately nourished and hydrated. Among 11 Polynesian plants, SO has significant promise for use as anti-aging agents, hair growth stimulants, and whitening treatments [18]. This study provides comprehensive information on all facets of Sigesbeckiae Herba, including taxonomy, ethnomedicinal uses, phytochemistry, and pharmacological properties, spanning the years 1980 to 2024. This is, to our knowledge, the inaugural review of SH, including a systematic detection and scientometric assessment of the extant studies. Scientometric assessment is a scientific tool to integrate statistical and mathematical techniques for further analysis of structures, trajectories, and objectives pertaining to a certain topic [19, 20]. Despite its widespread application in medicine and health, Scientometric analysis of SH is not documented yet, to the best of our knowledge [21]. Thus, this work aims at Scientometric assessment of SH -related literature applying the Scientometric program "VOS viewer". This comprehensive work, spanning several decades of research, offers critical insights and implications for funders, regulators, and researchers in herbal medicine, natural products, and drug discovery, ultimately guiding future advancements in the treatment of diverse inflammatory conditions. This knowledge may enable researchers globally to study unexamined species of the genus by extracting novel bioactive phytoconstituents to treat numerous disorders that remain uncured with minimal side effects.

### Research methodology

Web of Science was the search tool to screen the literatures from 1980 to 2024 containing the Scientometric analysis on SH**.** Chemdraw 19.0 was employed to draw the chemical structure and the plants/species scientific names were verified using plants of the world online: Royal Botanic Garden KEW (Plants of the world online).

### Data acquisition

This study gathered material relevant to SH from the Web of Science (WOS) core collection database on October 6, 2024. A search of SH-related scientific literatures was conducted applying the following terms: “Sigesbeckiae Herba, *Sigesbeckia* L., Xi-Xian Cao, Saint Paul's wort, *Sigesbeckia pubescens, Sigesbeckia glabrescens, Sigesbeckia orientalis.”* Literature published between 1980 and 2024 was included. The analysis included a diverse range of document types such as original research articles, review papers, editorials, meeting abstracts, conference proceedings, letters, book chapters, and early access publications. The final dataset was counting to total of 215 approved records that can be acquired by Scientometric analysis, then loaded into VOS viewer, and analysed for several characteristics, including co-occurrence, co-citation, citation, and co-authorship [22]. Keywords, Author Keywords, Abstract, and Title were considered the Term Source. The analytical findings were acquired and displayed as network maps and a graph in this study.

### Scientometric thematic analysis

In 1969, Nalimov and Mulchenko coined “Scientometric” as “a measurement of science.” Scientific metric study began since the nineteenth century and until now. Scientometric analysis has evolved from unconscious to awareness, from just qualitative tool to thoroughly examine scientific production's underlying qualities [23, 24]. Scientometric has shown its power in carbon footprints [25], computer vision in construction [26], cloud computing [27], construction and demolition waste [28], leak detection and localisation [29], off-site construction [30], oral health literacy [31], public–private partnerships [30], recommendation science [32], smart city [33], sustainability [34], unfrozen soil water [33], and virtual reality [35]. Using literature reviews, current Scientometric analysis may structure the updated knowledge, highlight the scientific contributions, locate scientific progress, and detect new advancements at the same time.

There is literature available on the *Sigesbeckia L*. genus that shows its diversity. Despite the fact of the broader exploration of the research area and better understanding, the risk of a biased limited interpretation is not yet excluded [36]. Thus, a thorough screening of the previous SH results applying a recent Scientometric is warranted. The method included countries/regions activities, authorship analysis, keyword co-occurrence analysis, literature coupling analysis, publications analysis, where VOS Viewer was applied as the visualisation and network modelling open-source software [21]. Notably, this research topic was generated by coupling the keywords with the literature analysis to create a scientific map. Subsequently, this systemic review was designed to complement the Scientometric analysis, where the applications and technologies were first investigated then the review structure was determined following this classification to disclose in-depth discussion.

## Results

### Publication type analysis

**Fig. 1:**
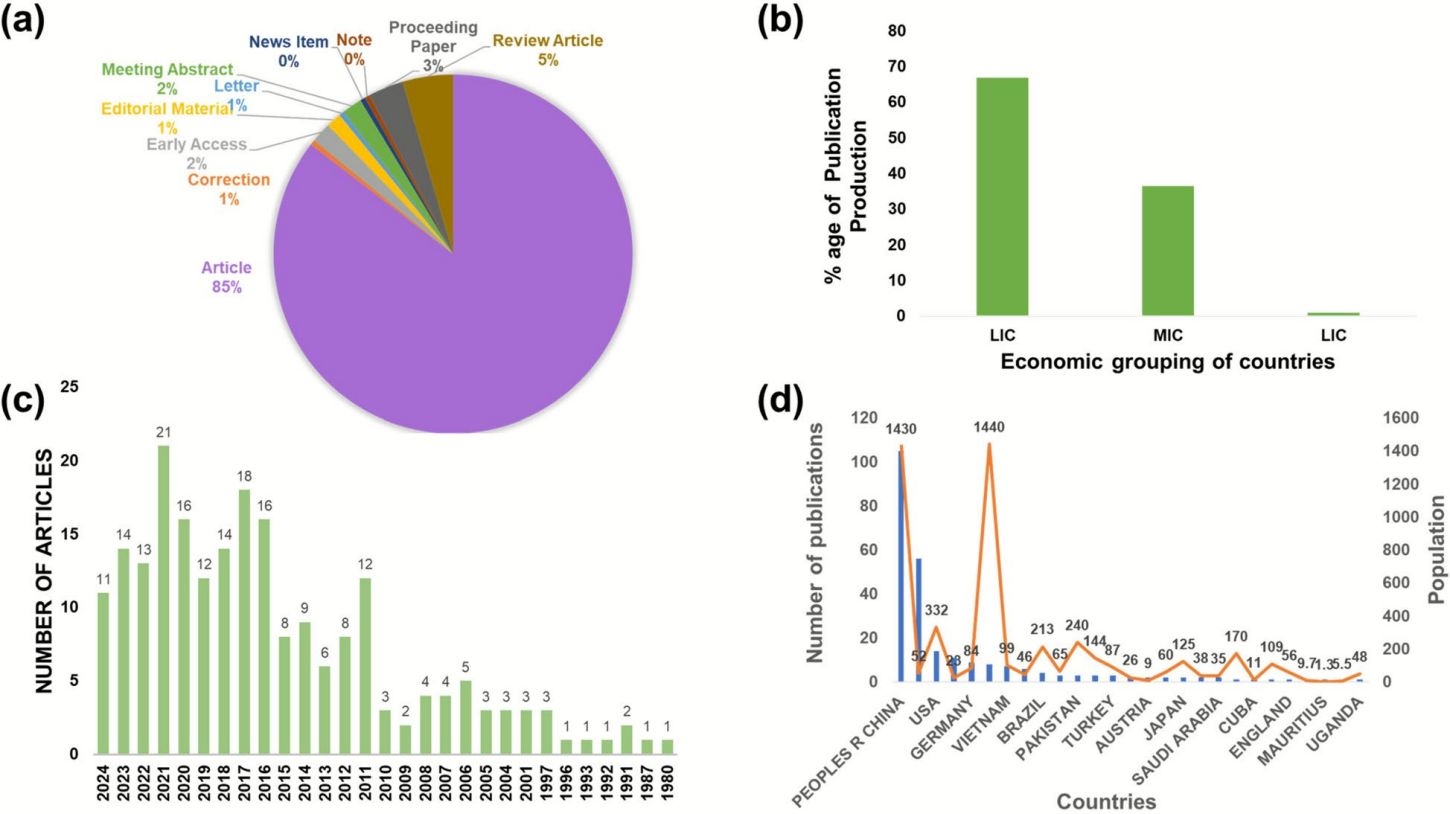
**a **Types of documents published on SH. **b **Publication production percentage on SH by different countries with respect to the economy, as per the World Bank. **c** Annual publications from 1980 to 2024. **d** 14 countries with higher publications regarding SH and population

Over the study period, meeting abstracts (1.86%) presented the lowest publications rate followed by review articles (4.651%), whereas preclinical articles were the highest (87.442%) as seen in Fig. 1. Screening the production of publications by the World Bank division took into account the economic status of the countries [37]. The proportion of the production with respect to the high-income, middle-income, and low-income countries was divided into 66.80%, 36.51%, 0.825%, respectively, as per SH and as illustrated in Fig. 1**.**

### Publications yield based on yearly analysis

Figure 1 portrays the annual publications ´occurrence from 1980 to 2024. Before 1980 there are no publications on SH on Web of Science. However, from 1980 to 2000 a total of 10 articles were published. This shows that gradually and slowly, people got awareness regarding this potential herb, which has many folkloric medicinal uses. From 2001 to 2010 almost 3 paper yearly are found on database. From 2011 onwards people started unveiling its merits. In 2021, more than 20 papers were published on this very herb. Taken together, the increasing number of publications on SH particularly its use in rheumatoid arthritis reflects a growing scientific interest in its pharmacological relevance. While this scientometric review does not assess therapeutic efficacy directly, the bibliometric trend suggests that SH is gaining attention as a candidate for further exploration. Therefore, we interpret this upward trajectory as a signal of emerging research momentum and unmet investigative depth, justifying the call for more in-depth studies to uncover its broader biomedical potential.

### Co-author’s organizations and publication analysis

**Fig. 2:**
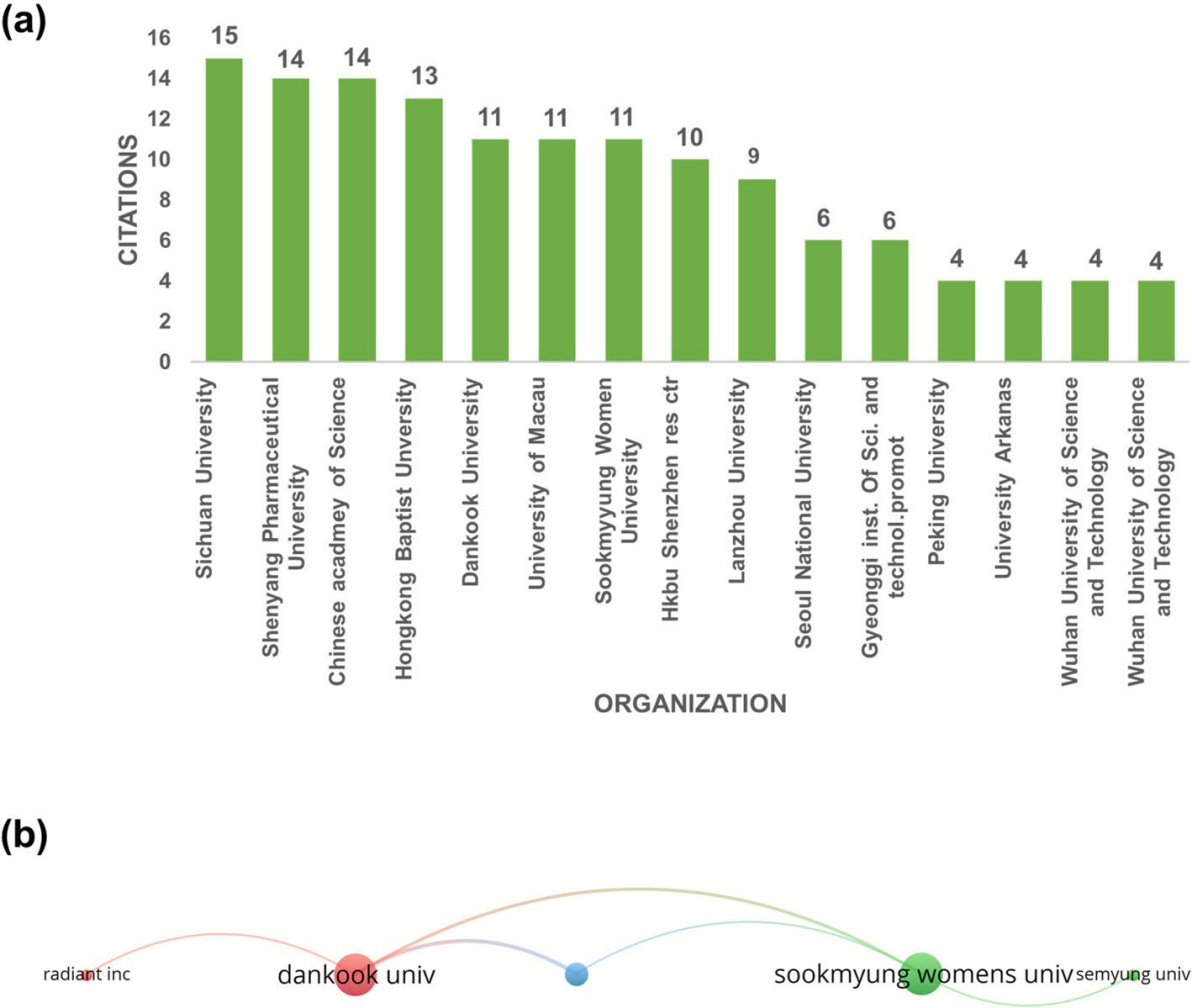
(**a**) Co-authorship Organization analysis globally regarding SH publication production. **b** Network of the top collaborating organizations globally

Further, the analysis touches on the organization’s collaboration, where 270 organizations in total were included in the SH -related research**. **As illustrated in Fig. 2(a), Sichuan University and the Chinese Academy of Sciences have the highest number of publications (N = 15), followed by Shenyang University (N = 14), Hong Kong Baptist University (N = 13), and Dankook University, Sookmyung Women's University, and the University of Macau (N = 11). Each node represents the organization/affiliation shown in our data od Co-authorship analysis-organization. For example, here Dankook University, 3 links, 14 link strength and 11 documents; Sookmyung Women's University, 3 links, 11 link strength, 11 documents; Semyung University, 1 link, 3 documents, 3 link strength; Gyeonggi University of Science and Technology, 2 links, 9 link strength and 6 documents; or Radiant inc., 1 link, 3 link strength and 33 documents. The size is showing that how often it occurs or how strongly it is connected (a bigger node means a greater number of documents. A thicker/stronger line generally means a stronger or more frequent co-occurrence, while a thinner line means a not frequent collaboration.

A network map of the findings is seen in Fig. 2 (b). This map was grouped into 3 clusters, where each cluster was radiated by multiple organisations. The Hongkong Baptist University, and university of Macau Collaborated with other organizations.

### Author co-authorship and the publication proportion

**Fig. 3:**
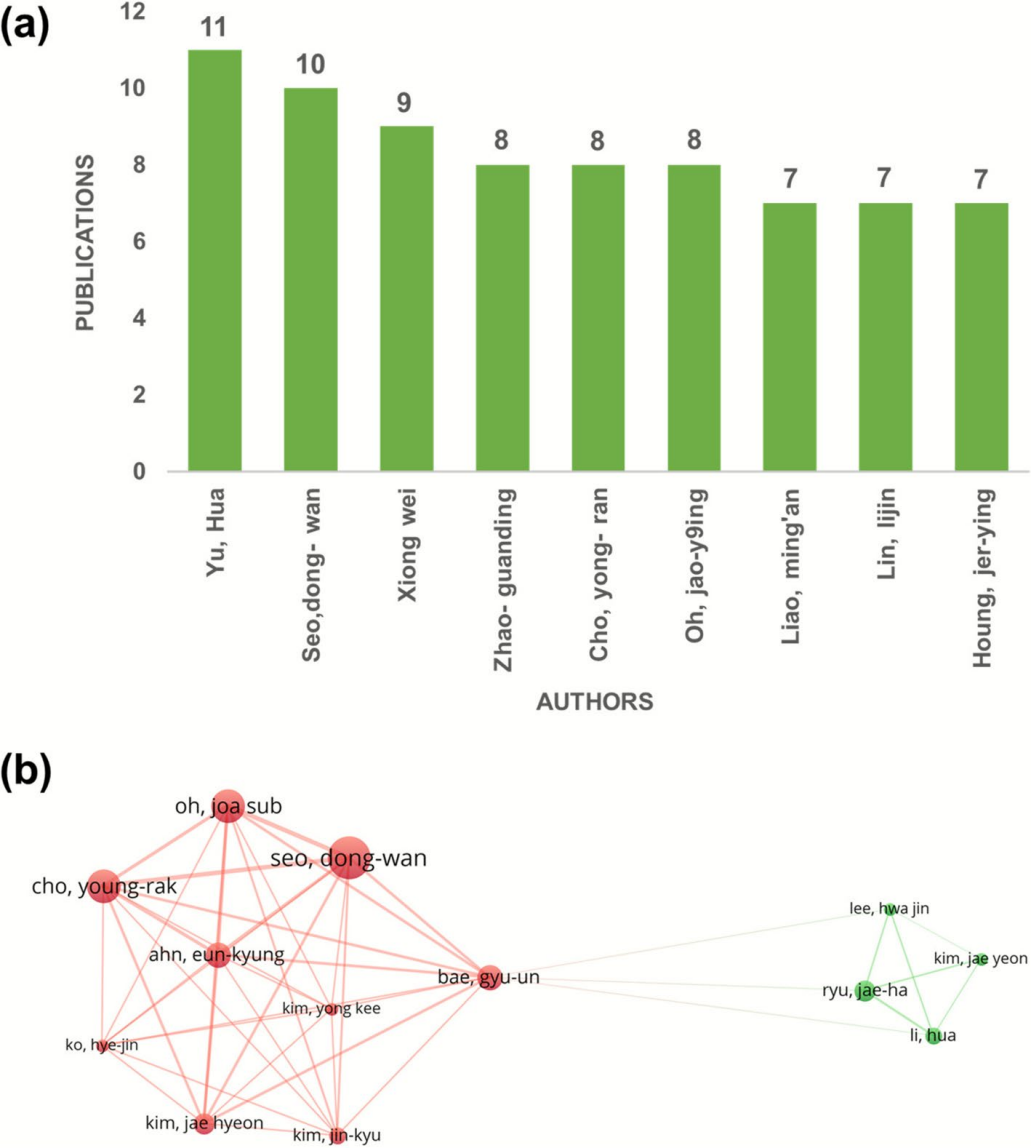
(**a**) Prominent Coauthor-Authors vis-à-vis SH publications (**b**) Network of author co-authorship analysis

The level of contribution in research can be evaluated in accordance to the authors production, typically measured by the total number of academic publications [25]. Accordingly, the topmost authors and their research contributions are displayed in the bar graph of Fig. 3a. Among the top list are Yu Hua then Seo dong-wan followed by Xiong Wei contributing by 11, 10 and 9 publications, respectively. But Seo dong-wan with the biggest node with 10 documents, 42 link strength and 8 links in total. Bae Syu-un showed the maximum number of links 1, with 6 documents and 37 link strengths that shows its impact. Cho young-rak has 8 links and 39 link strength with 8 documents. A thicker or darker line usually means they have published together more often (stronger link strength), while a very thin line means only a few shared papers. The size of the circle usually shows how many co-authored papers that author has (bigger circle = more collaborations/higher total link strength). The collaboration between co-authors was described as a network map as seen in Fig. 3b. Similarly, 80 authors meet the threshold, and out of that, 13 are connected and appear as two clusters. Cluster 1 has 9 items, all have 8 links, and one of them has 11 Bae gyu-un and cluster 2 has 3–4 links. It is anticipated that future endeavours in research related to SH, will result in a better collaboration.

### Publication analysis of co-author's countries

**Fig. 4:**
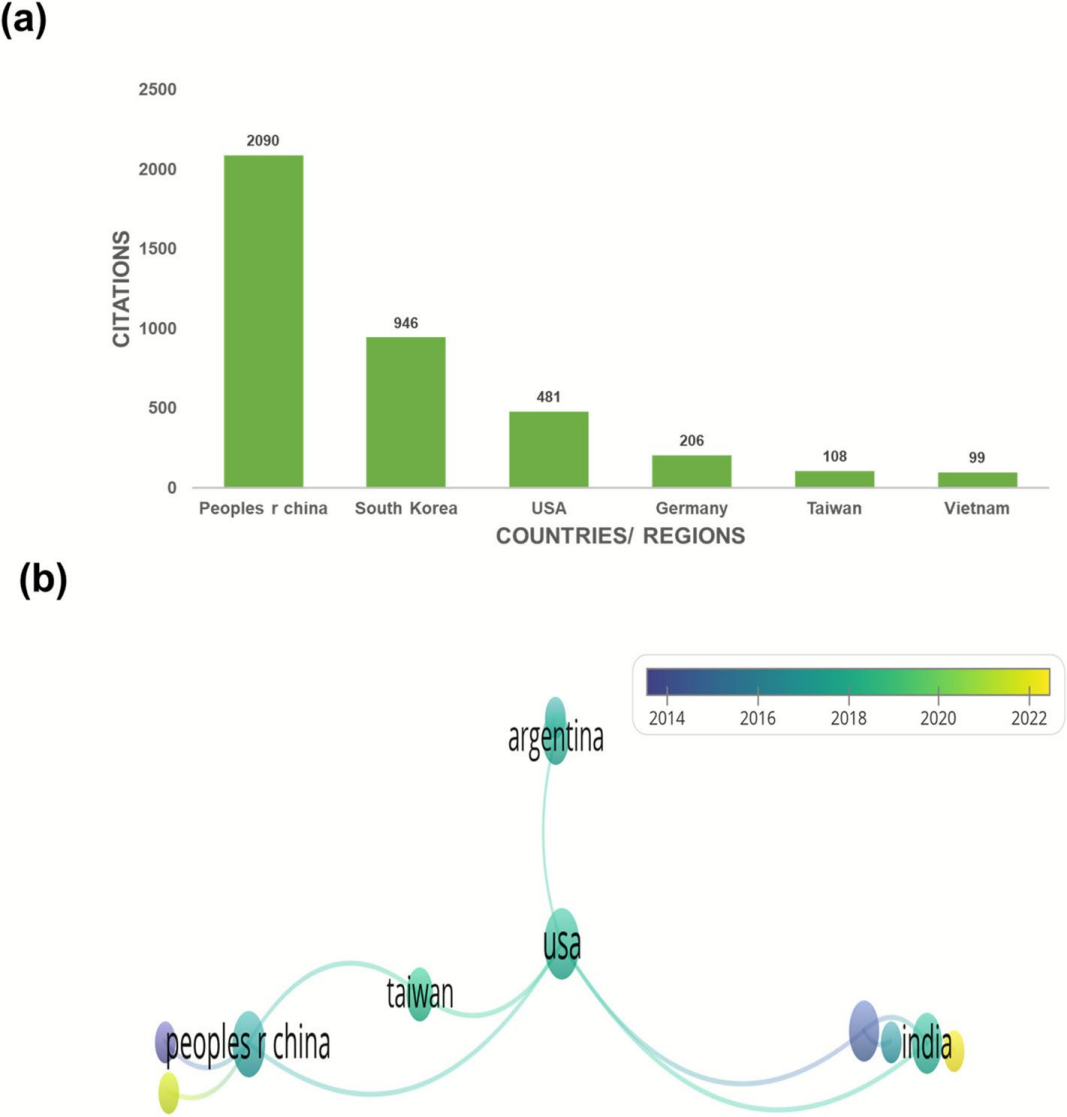
(**a**) Co-authorship analysis among countries contributing to Sigesbeckia herba research. (**b**) Overlay of Co authorship analysis of countries

People’s Republic of China has the highest number of documents (103) on SH to date, which is due to the long history of application and wide investigation in China. The fewest publications (N = 1) were reported from Bangladesh, Cuba, Egypt, England, Hungary, Mauritius, Norway, and Uganda. Undoubtedly China is playing a key role in putting effort in making nature to cure ailments. It is at first number with 47.906% of the record followed by South Korea (N = 55) 25.581% and USA (N = 14) 6.511%. articles posed by remains countries are 38. The data clearly indicate that substantial research opportunities remain in the study of Sigesbeckia herba. The collaboration network was divided into five distinct clusters, each primarily led by one or two countries. USA has the maximum links with other countries, which is 5, i.e., Argentina, USA, South Korea, India, and Taiwan shown in Fig. 4.

While publication volume is highest in China, citation impact varies across countries. This suggests that international collaborations—such as those led by the USA may contribute to greater visibility and influence, reinforcing the importance of co-authorship networks.

### Citation analysis

**Fig. 5:**
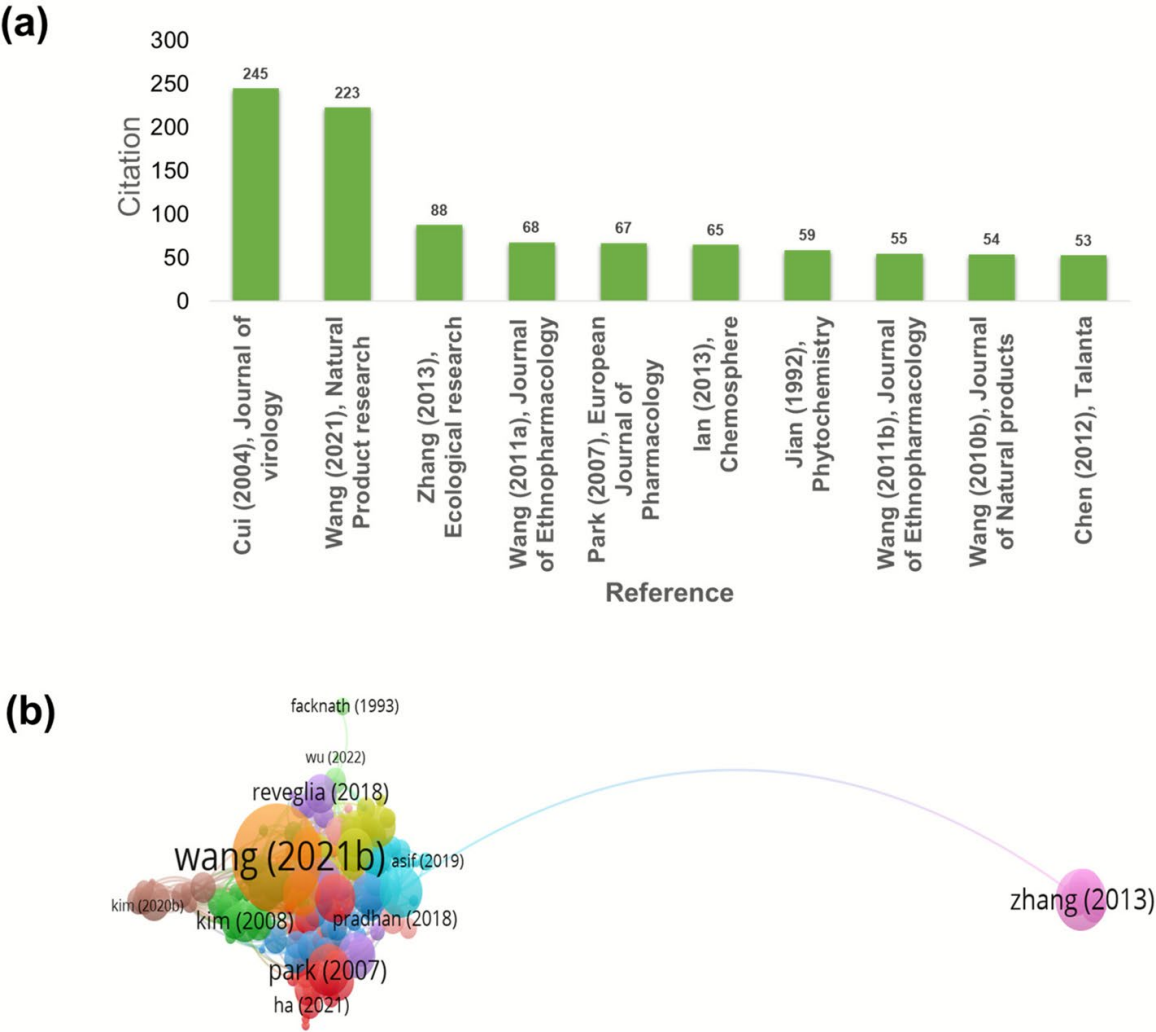
(**a**) Bar graph showing documents cited the most. (**b**) Network of document/publication cited frequently

**Fig. 6:**
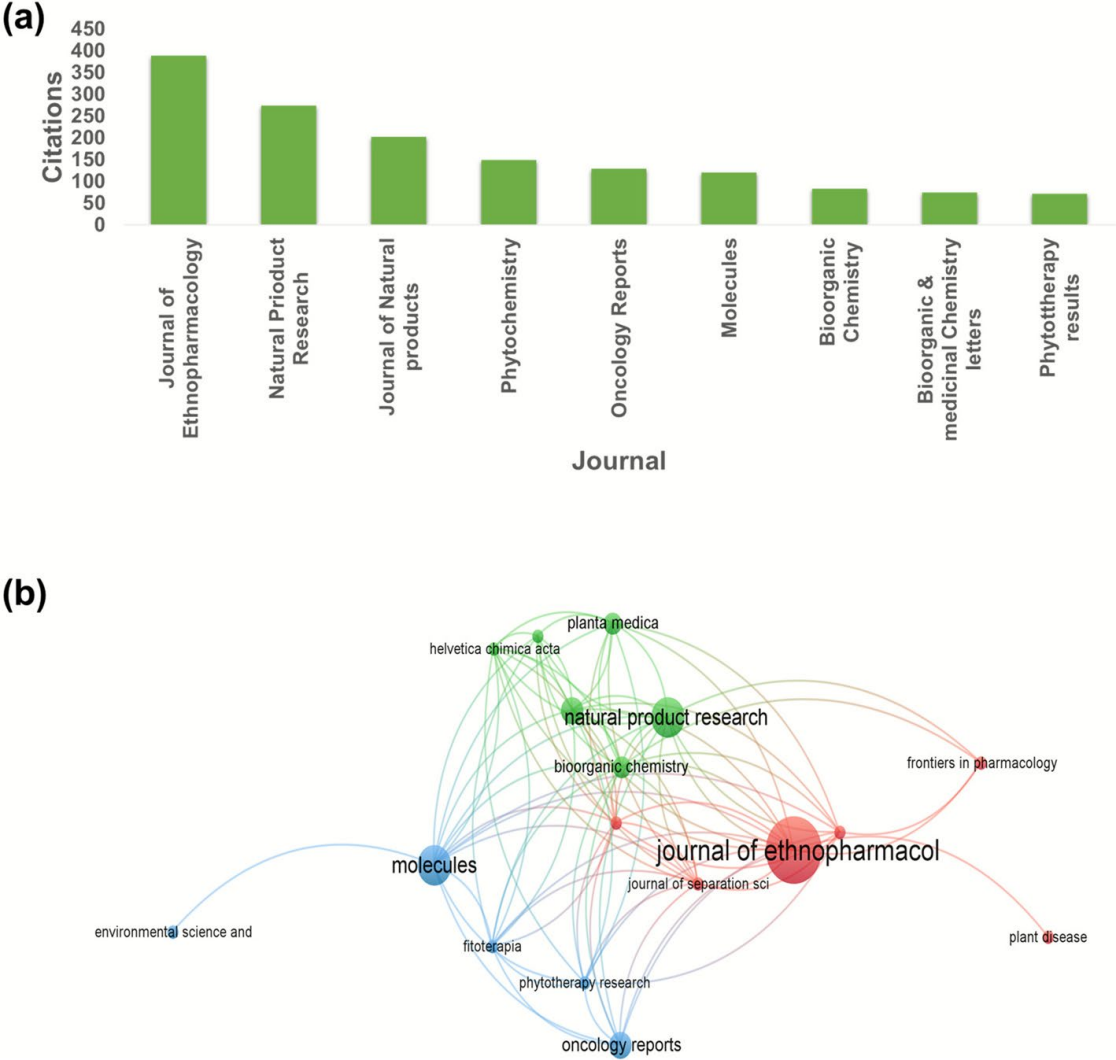
(**a**) Journals with high number of citations. (**b**) Citation and sources network of 17 journals

**Fig. 7:**
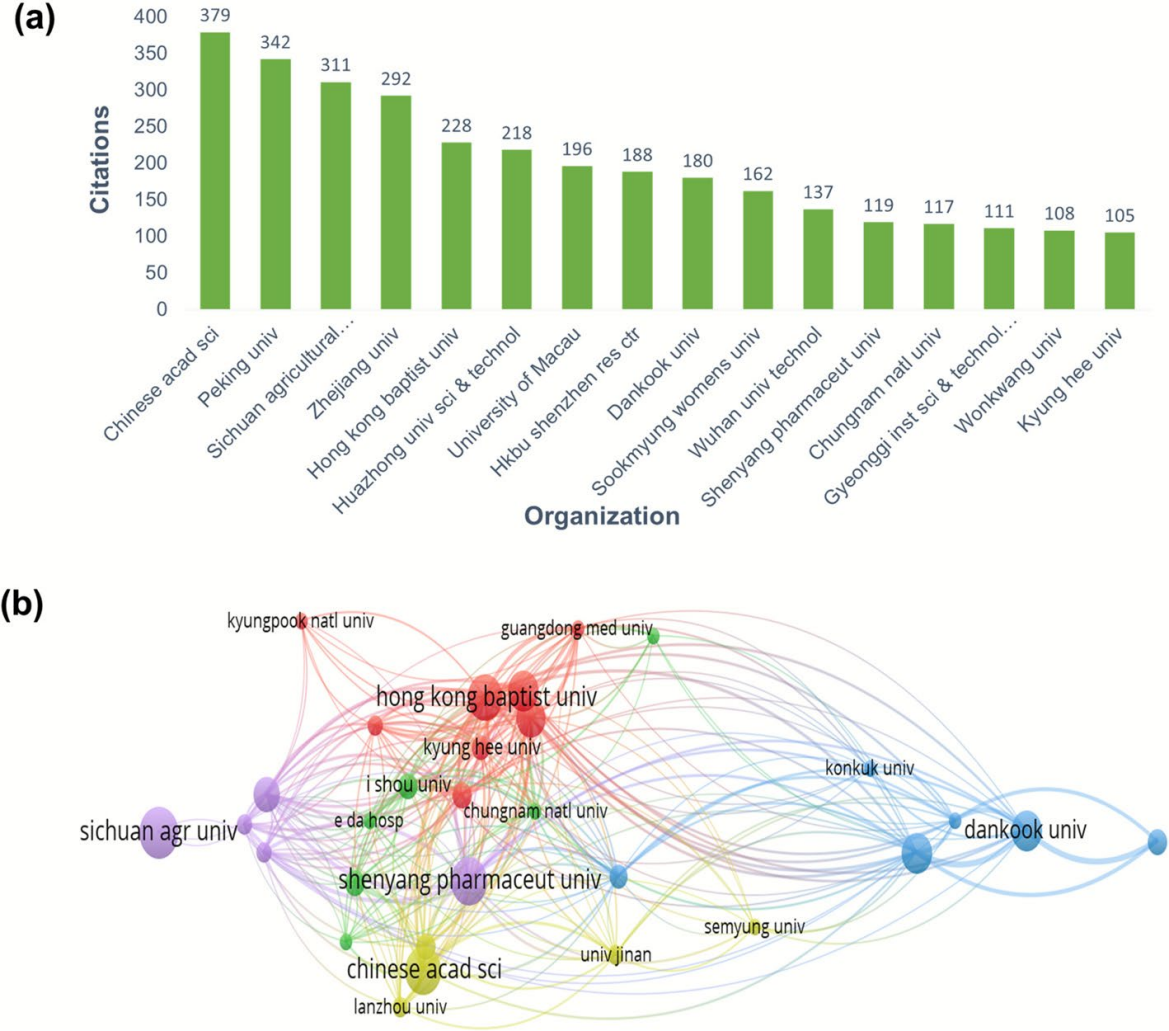
(**a**) Top 16 organizations with high citations, (**b**) Citation network visualisation of 33 Universities

**Fig. 8:**
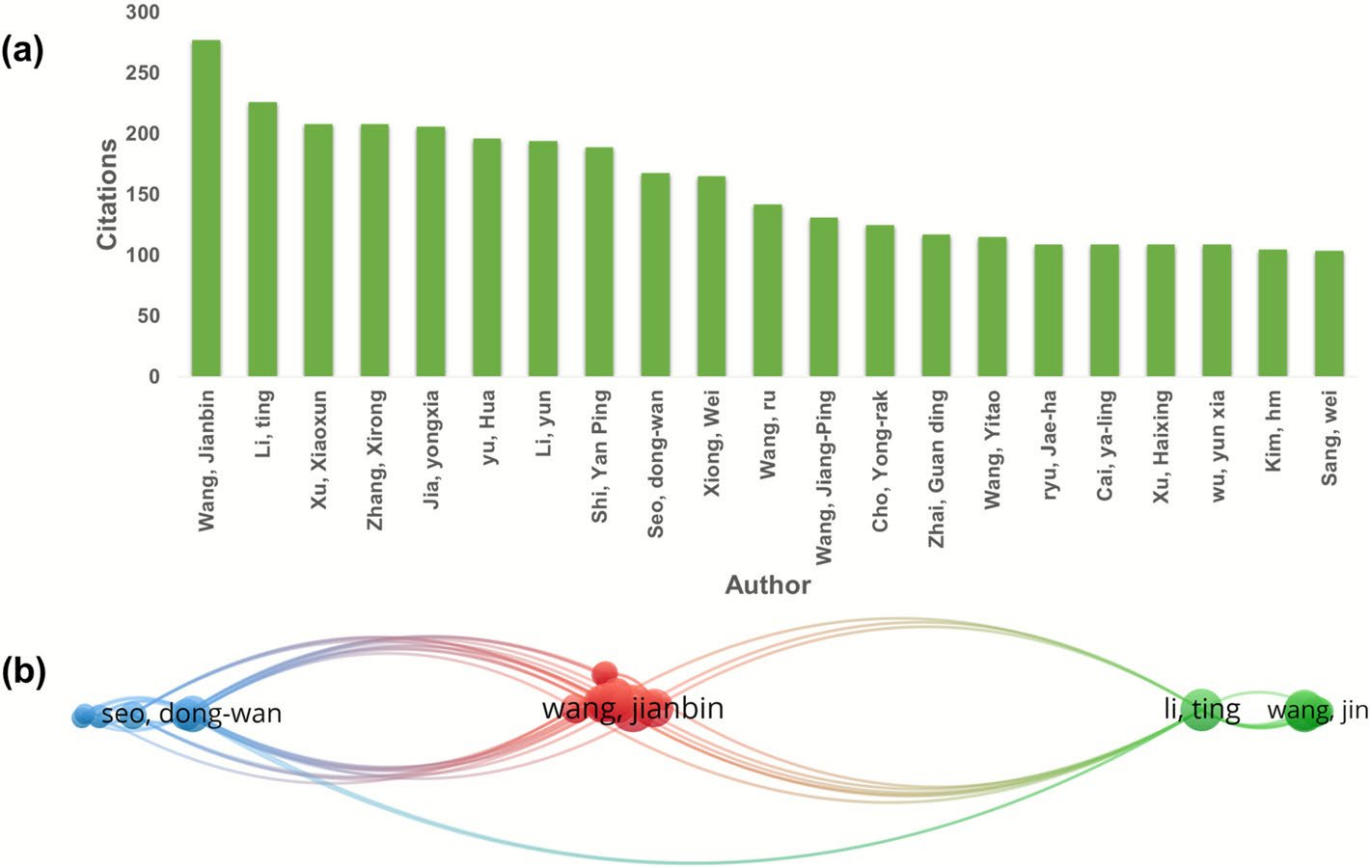
(**a**) Authors with high citations. (**b**) Network of authors with high citations

Publications/countries/organizations/journals citation analysis are described in a previous report from VOS viewer. Figure 5a where the Chinese academy of Science, Chungnam national university, Zhejiang University, Wuhan university of science and technology, Wuhan university of technology, Sichuan agricultural university, Huazhong University of science and technology, GYEONGGI inst. science & technology promotion, Wonkwang university, sookmyung women university are cited for 379, 342, 311, 292, 228,218 respectively regarding research on herba Sigesbeckia. Taken together, China, Korea and USA were cited 2056, 942 and 496 times respectively and thus placed first, 2nd and 3rd in order. Figure 5b shows the network visualisation of 33 Universities. As shown in Fig. 6a & b, Cui (2004) in the Journal of Virology, Wang (2021) in Natural product research and Zhang (2013) Ecological research were the top three highly cited publications (245, 223, 88 citations, respectively). Metronomic cyclophosphamide regimen, anti-angiogenic basis of metronomic chemotherapy, and tumour angiogenesis are the research areas that attract relatively more attention, where 50 citations were reported in relation to 7 articles in this line. This is still promising for an herb that has recently been getting serious attention. Figure 6b. Among the leading journals contributing to Sigesbeckia research, the Journal of Ethnopharmacology stands out with 15 publications and 389 citations. Molecules and Natural Product Research each contributed 8 articles, receiving 203 and 274 citations, respectively. The analysis method was developed based on the threshold and the citations (so at least 5 documents and 5 citations) to create a network map, so out of 130, 16 meet the thre*sh*old. Figure 7a. In this way, the selection of the most suitable journals to publish research in areas of herbs and plant-based strategies becomes easier. Considering the citation of documents related to SH Peoples republic of China is leading the list with maximum citations to date which is 2056 shown in Fig. 8a**.** Followed by south Korea an USA with 942 and 476 Citations. Out of 26 countries 13 meet the threshold. Authors who have been highly cited through all this time on research on SH, Wang Jianbin from Peking University, Beijing, particularly School of Pharmaceutical Sciences, State Key Laboratory of Natural and Biomimetic Drugs has the maximum number of citations on the research on herba Sigesbeckia.

### Co citation analysis

Article´s citations would enlist the papers contributed to the field and inform about the status of research and the latest of knowledge in this regard. The analysis of the co-citation relationship can provide enough bibliometric information about the active authors, the research frontiers, and the directions of mutual exchange, thus the number of times they are co-referenced are the base for co-citation analysis.

**Fig. 9:**
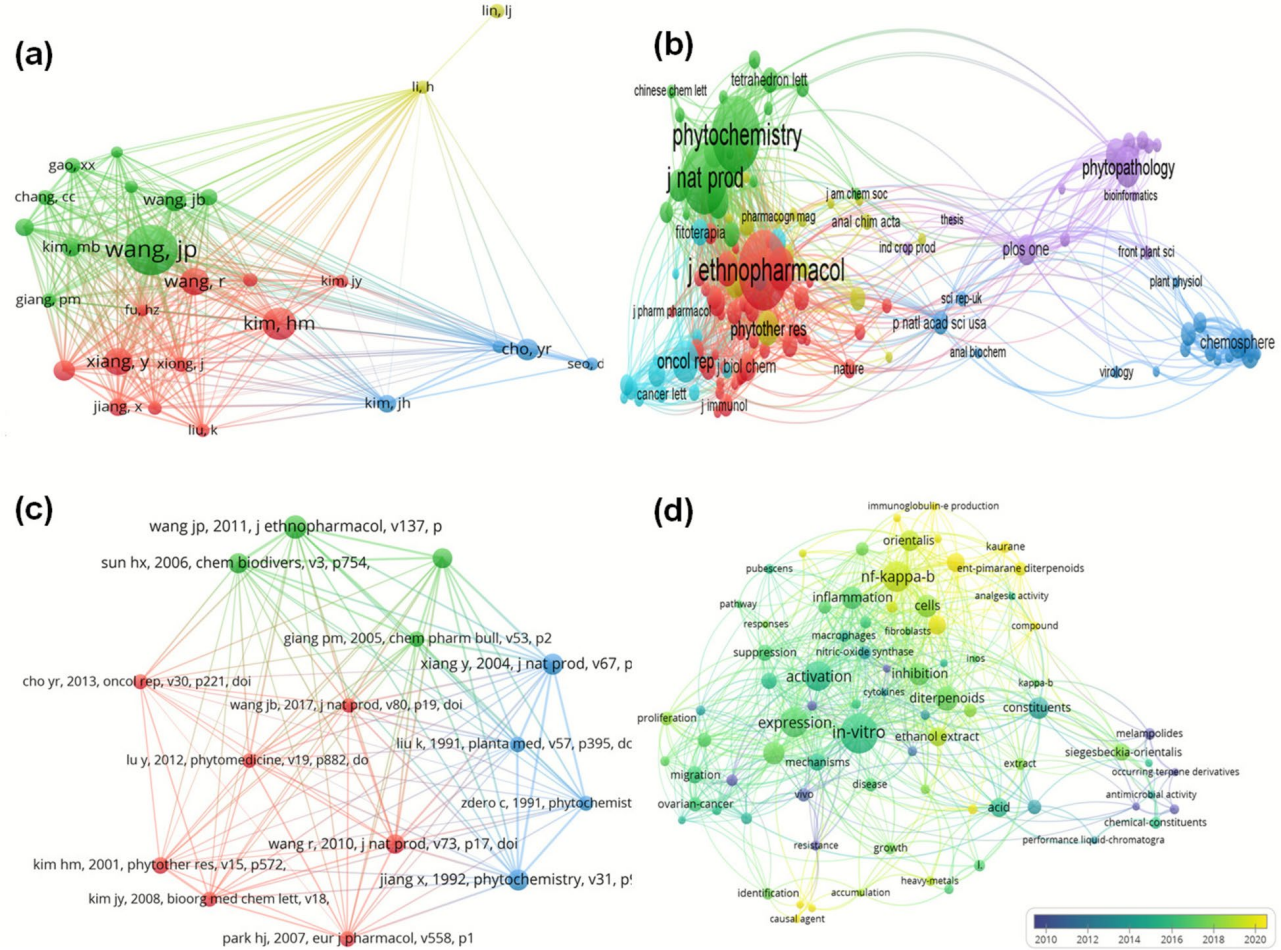
(**a**) Co citation and author analysis network. (**b**) Network showing the journal with more citations (**c**) Network map for reference- co citation (**d**) Represents the Keyword Concurrence Analysis, a visual representation of our study's findings

A total of 4761 authors were obtained in 241 publications. In Fig. 9a, it is evident that the top three authors are Wang jp, Kim Hm, Wang R., and Wang Y. with the greatest citations number i.e., 89, 57 and 48 respectively regarding research on herba Sigesbeckia. 10 authors are cited more than 30 times till date on research carried out on SH. The authors/journals/references relationship was identified by co-citation analysis based on their citation on every third document [37]**.**

The co-cited journals/sources phytochemistry, Journal of natural products, Journal of ethnopharmacology, Molecule and phytopathology are cited the most for research related to SH. They have citations 234, 207, 91,88, 84, respectively. As the WOS data shows that there is still a big room left for further research on SH so we cannot find citation in thousands. The co-citation network with the source/journal can give better direction to the authors for selection of appropriate and reasonable journal if it comes to some data/research related to herbs. Co-citation is also analysed with cited references.

It is popular to refer to reputable journals that is well-known and highly cited sites as they promote the innovative ideas and unusual findings. In this section Fig. 9b is illustrating the relationship between collaboration and co-reference where the influential publication that left impact or raised attention were on the top list. [10, 13, 38–42] publications were the prominent in the Journal of Ethnopharmacology, phytochemistry and Journal of Natural products and displayed high nodes magnitude in Fig. 9b. The other mentioned references have also been cited at least more than 20 times.

References as well as keywords, are usually the vital tool to identify the research footsteps as it reflects directly and indirectly the research subject. Hence, keyword occurrence analysis is a key player when looking after the study line, content and context in a significant way cannot be overstated. Through this method, researchers can effectively summarise the critical points, thereby reflecting the topic of the research. Therefore, keyword co-occurrence analysis is not just a tool, but a crucial method for defining the main study themes and preferred word combinations [20]**.**

215 papers were collected, and the keywords were meticulously retrieved ensuring a comprehensive analysis in this study, where 130 words out of them were used to create an overlay map for clarity and visualization. The 130 keywords that met the threshold with at least 5 occurrences minimum, demonstrating the thoroughness of the research show in Fig. 9c. The node size in the map is representing the number of times the keyword appears, providing a clear visual representation of the data. The coloured nodes are proportional to the average year of keywords emergence, further enhancing the clarity of the findings. In addition to keyword inhibition, inflammation, NFκ-β, diterpenoids, Kirenol, *S.* orientalis, *S.* glabrescens, *S.* Pubescens and invitro appear frequently. The keywords that appear in yellow represent the research direction for recent years, such as Rheumatoid arthritis, Collagen-induced arthritis, Molecular docking, and *En*t- pimaerane diterpenoids, *S. Pubescens*, NF-κβ. In VOS viewer, the number of linkages of a keyword with other is seen as link strength and the occurrence weight by the size of a given keyword. Figure 9d represents the keyword concurrence analysis, a visual representation of our study's findings.

### Targeted literature review of phytochemicals and bioactivities

Parallel to the scientometric analysis, we performed a targeted literature study not adhering to a formal systematic review protocol to investigate the phytochemical profile and pharmacological properties of three species of Sigesbeckiae Herba (*S. orientalis*, *S. pubescens*, and *S. glabrescens*). We conducted a search in PubMed, Scopus, and Web of Science utilising combinations of keywords including “Sigesbeckia,” “phytochemicals,” “bioactivity,” “anti-inflammatory,” “antioxidant,” and “traditional medicine.” The inclusion criteria were: (i) original research publications published in English; (ii) studies detailing specific phytochemicals or bioactivities of the chosen Sigesbeckia species; and (iii) articles published from 2000 to 2024. The exclusion criteria comprised: (i) review articles, editorials, or commentaries; (ii) research devoid of species-level identification; and (iii) publications with inadequate experimental description. This methodology enabled us to carefully consolidate the existing information regarding the bioactive chemicals and therapeutic potentials of Sigesbeckia herba, while complementing the scientometric analysis of research trends.

**Fig. 10:**
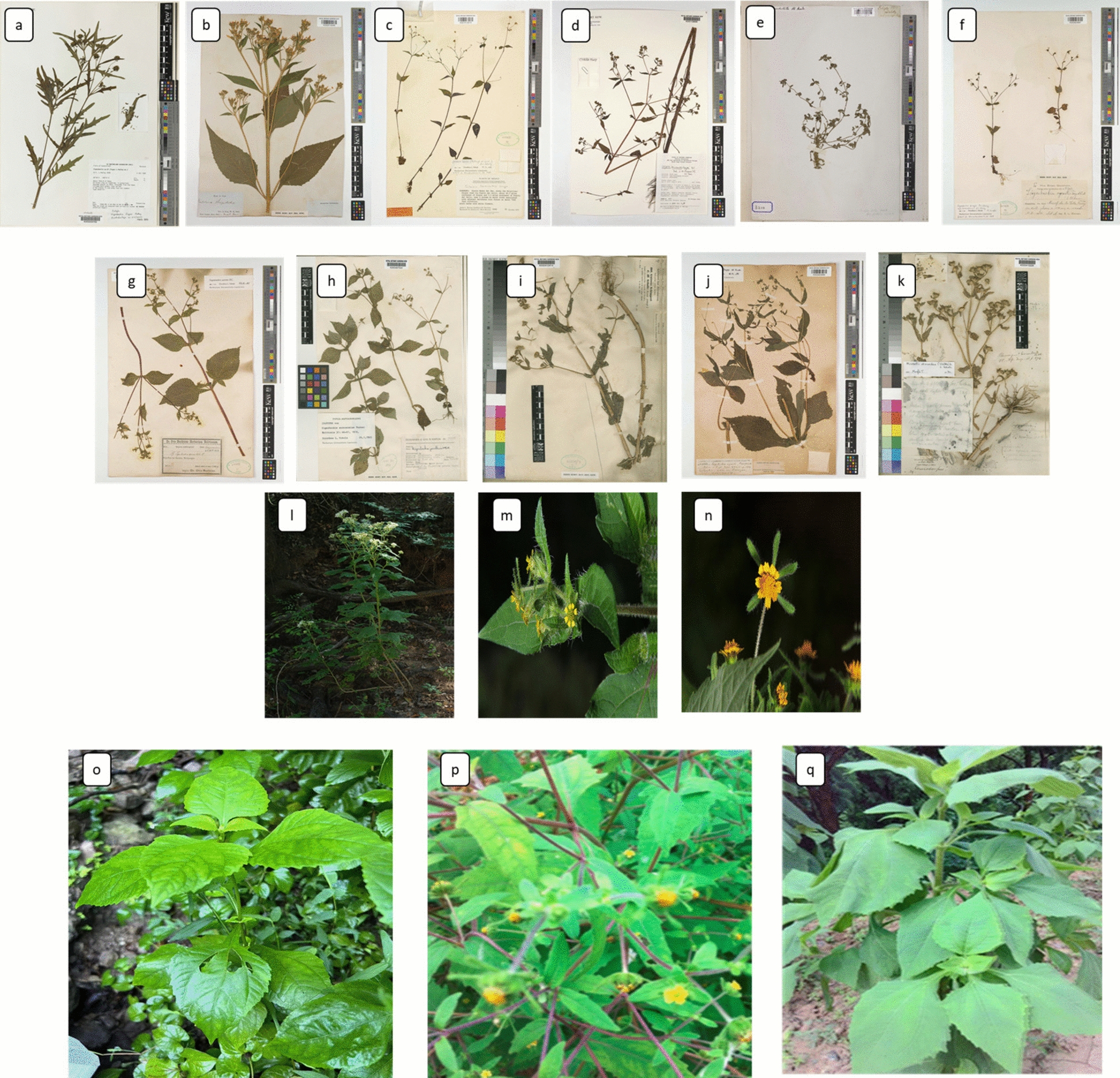
Representative pictures showing morphology of **a**. *Sigesbeckia australiensis* subsp. *fugax* (Pedley) Orchard | Plants of the World Online | Kew Science, accessed on 20–09–2024; **b**. *Verbesina occidentalis* Walter | Plants of the World Online | Kew Science, accessed on 20–09–2024; **c**. *Sigesbeckia blakei* [44] accessed on 20–09–2024; **d**. *Sigesbeckia bojeri* (DC.) Humbert | Plants of the World Online | Kew Science accessed on20-09–2024; **e**. *Sigesbeckia portoriccensis* Bertero ex DC. | Plants of the World Online | Kew Science accessed on 20–09–2024; **f**. *Sigesbeckia pringlei* D.L.Schulz | Plants of the World Online | Kew Science accessed on 20–09–2024; **g**. *Sigesbeckia mandonii* Sch.Bip. | Plants of the World Online | Kew Science accessed on 20–09–2024; **h**. *Sigesbeckia andersoniae* B.L.Turner | Plants of the World Online | Kew Science accessed on 20–09–2024; **i**. S*igesbeckia somalensis* S.Moore | Plants of the World Online | Kew Science accessed on 20–09–2024; **j**. *Sigesbeckia flosculosa* L'Hér. | Plants of the World Online | Kew Science, accessed on 21–09–2024; **k**. *Sigesbeckia discoidea S.F.Blake | Plants of the World Online | Kew Science* accessed on 21–09–2024; **l**. *Sigesbeckia laciniat*a Poir. | Plants of the World Online | Kew Science accessed on 21–09–2024; **m***. Sigesbeckia jorullensis* Kunth | Plants of the World Online | Kew Science accessed on 21–09–2024; **n**. *Sigesbeckia agrestis* Poepp. | Plants of the World Online | Kew Science accessed on 21–09–2024; **o**. *Sigesbeckia glabrescens* Makino | photo was taken by Dr. Yu at University of Macau; **p**. *Sigesbeckia orientalis* L. | photo was taken by Dr. Yu at University of Macau; **q**. *Sigesbeckia Pubescens* Makino | photo was taken by Dr. Yu at University of Macau

### Classification

Only 17 species of *Sigesbeckia* L., genus are presented here with approved names and pictorial references. The focused main species which have been explored to some extent i.e., *S. orientalis* synonyms *S. brachiata* Roxb., *S. droseroides* Sweet, *S. caspia* Fisch. & C.A. Mey, *S. esquirolii* H. Lév. & Vaniot., *S. glutinosa* Wall., *S. gracilis* DC., *S. humilis* Koidz., *S. abyssinica* Oliv. & Hiern, *S. microcephala* DC., *S. triangularis* Cav., *S. orientalis* var. *angustifolia* (Makino) Makino, *S. orientalis* var. *tenggerensis* Hochr., *S. orientalis* f. *angustifolia* Makino, *S. pubescens* synonyms *S. orientalis* var. *pubescens* (Makino) Makino, *S. orientalis* f. *pubescens*, *S. pubescens* f. *eglandulosa* Y. Ling & Hwang and, *S. glabrescens* synonyms, *S. formosana* Kitam., *S. glabrescens* var. *leucoclada* Nakai, *S. orientalis* var. *glabrescens* (Makino) Makino, *S. pubescens* var. *glabrescens* (Makino) Vorosch. *S. orientalis L.*, *S. glabrescens* L. and *S. pubescens* makino are the three among this genus used in research traditionally and currently (Fig. 10) [43]

### Morphology and distribution

**Fig. 11:**
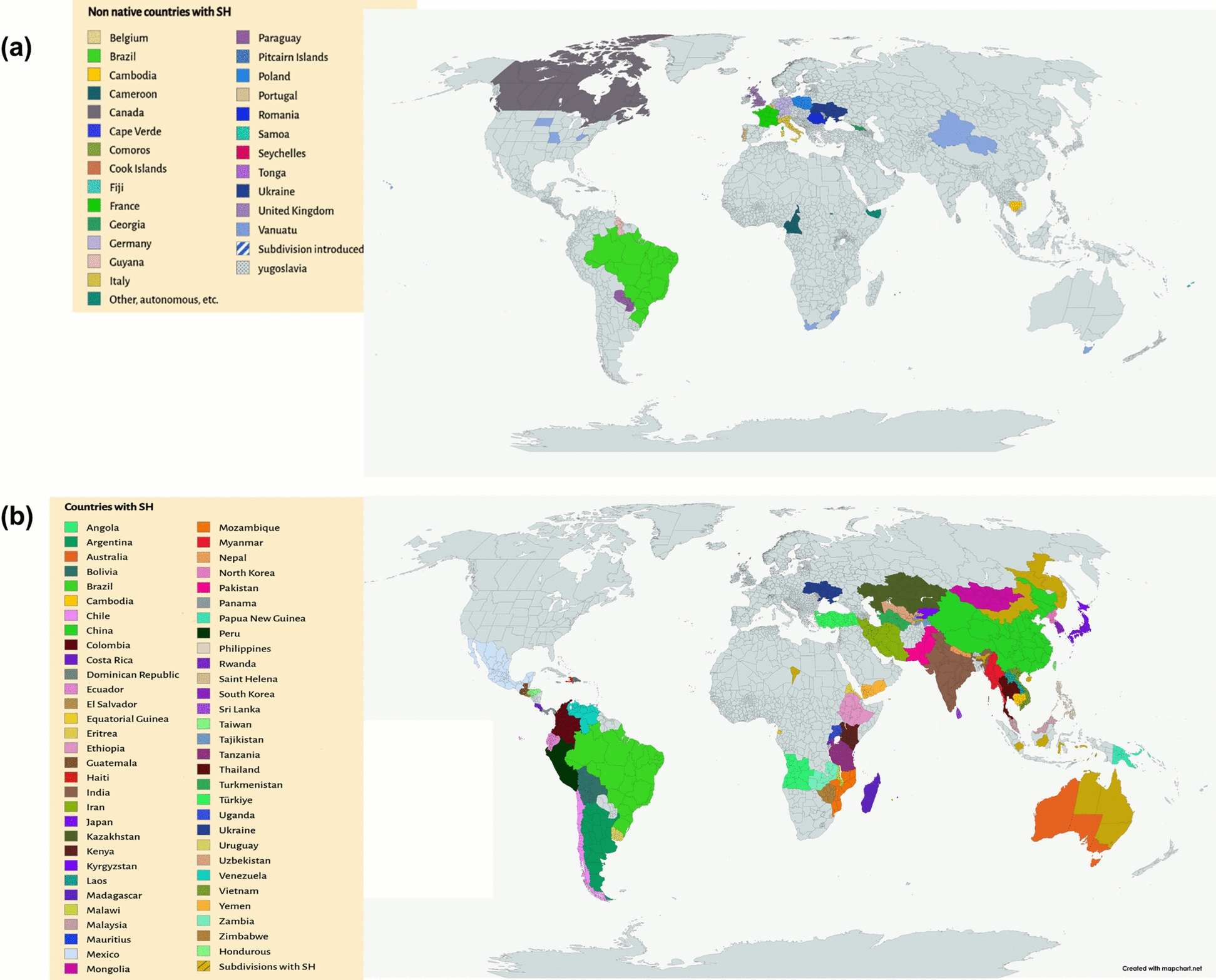
(**a**) Regions of world SH is introduced. (**b**) Indicative of its native habitat of SH. The graph is made from mapchart.com [49]

The morphology and geographical distribution of SH are presented below in Table 1 and Fig. 11, which reports their floral habitat harvesting season and structural differences among the three main species [4]. Some variations in the stem’s shapes and height, sizes of the pedicel, the leaves, and other items are *sh*own.

**Table 1:** Morphological characteristics and geographical distribution of *Sigesbeckia orientalis L* (SO), *S. pubescens Makino* (SP) and *S. glabrescens Makino* (SG)

Vegetative structure	Floral habitat	Occurrence in world
*Sigesbeckia orientalis.* L (SO)		
Branches are dichotomous, having white -grey pubescenct. Leaves are Ovate triangular, ovate, or lanceolate-ovate, length of 2.5–11 cm and width of 1.5–7 cm, without veins pubescence Stem is Stout, brown purplish, with height of about 30–100 cm The capitulum diameter is 15–20 mm; with dense pedicel pubescence Florescence: April to September	Grows in mountains, grassy bu*sh*es, and woods at an altitude of 100–2700 m	Amur, Angola, Assam, Borneo, Cambodia, China South-Central, China North-Central, East Himalaya, China Southeast, Ethiopia, Eritrea, India, Hainan, Inner Mongolia, Japan, Iran, Kazak*hs*tan, Jawa, Khabarovsk, Kenya, Korea, Kirgizstan, Lesser Sunda Is., Laos, Malawi, Madagascar, Maluku, Malaya, Mauritius, Manchuria, Myanmar, Mozambique, Nepal, Nansei-*SH*oto, New South Wales, New Guinea, Northern Territory, North Caucasus, Pakistan, Philippines, Queensland, Primorye, Rwanda, Rodrigues, Réunion, South Australia, Socotra, Sulawesi, Sri Lanka, Tadzhikistan, Sumatera, Taiwan, Thailand, Tanzania, Transcaucasus, Tibet, Turkmenistan, Uganda, Turkey, Victoria, Uzbekistan, West Himalaya, Vietnam, Yemen, Western Australia, Zaïre, Zimbabwe, Zambia
*Sigesbeckia pubescence* Makino (SP)		
Branches are much-branching, grey, white hispid or villose Leaves are Orbicular-ovate located to the middle of the plant, while lanceolate to the upper; 3.5–12 cm tall and 1.8–6 cm wide; with veins villosa. Stout, green greyish, of 30–110 cm height Stem is the capitulum is of 18–22 mm diameter, with densely brown purplish glandular pedicel hairs. 60–120 cm tall densely covered with white pubescent Florescence: May to August	It grows on hillsides, valley forest margins, stream sides, wildness, and cultivated land at 160–3400 m	Tibet, Taiwan, Primorye, Manchuria, Korea, Khabarovsk, Japan, Inner Mongolia, Hainan, East Himalaya, China Southeast, China South-Central, China North-Central, Assam
*Sigesbeckia glabresence* Makino (SG)		
Branches are much-branching, white -grey flat pubescent Blade-ovate leaves; length of 2.5–11 cm and width of 1.5–7 cm; without veins villosa Slender, brown purplish Stem, and height of 30–100 cm The capitulum is of 10–18 mm diameter; has sparse pedicel has pubescence Florescence: April to September	growing on roadside, wildness, and hillside thickets at 300–1000 m altitude	China Southeast, China South-Central, Taiwan, Primorye, Nansei-*Shoto* islands

### Sigesbeckia tri-species bioactivity analysis

SH and its constituents were proven to have pharmacological activities, including anti-inflammation, anti-tumour, antithrombotic, antibacterial impact, and in the protection against cerebral ischemia. This evidence supports its clinical use in TCM for treating rheumatoid arthritis and cerebral ischemia. Network pharmacology also shows that Xi-xian Cao also known to refer to it as "loathsome harlotry" because of the focus of the system upon the presence of (or lack of) sex organs regulate various signalling pathways, including VEGFA-VEGFR2 and PI3 K-Akt, to promote vascular repair and regeneration, activate cell survival, and inhibit neuronal apoptosis. It also regulates lipid homeostasis and inflammation, prevents platelet aggregation, and plays an antithrombotic role. This study suggests that Xi-xian Cao is crucial in treating ischemic stroke by preventing thrombosis, promoting angiogenesis, protecting neurons, having anti-inflammatory effects, and regulating blood pressure and lipids, potentially developing as an anti-ageing, anti-blemish, and whitening element [19, 45–48]

The comprehensive summary of these pharmacological activities is presented in supplementary material Table 1, providing valuable insights into the potential benefits of Sigesbeckiae Herba*.*

### Anti-inflammatory

The ethanolic extract from aerial parts of SH at 4 mg/kg showed significant anti-inflammatory effects, reduced ankle joint inflammation, and provided analgesia with a 65% success rate in rat model of adjuvant- induced arthritis. It also improved T-lymphocyte proliferation, IL-2 activity, and IL-1 activity in comparison with the control (P < 0.01) [50]. Studies show that acetylsalicylic acid (100 mg/kg) and ent-16 α-H, 17-hydroxy-kauran-19-oic-acid (20 mg/kg) isolated from roots of *S. pubescens* have declined the inflammatory mediators including iNOS, COX-2, and TNF-α by reducing NF-κB blinding activity [51]. *S. orientalis* extract (aerial parts) at 4–5% (5 mL/day) decreased inflammation, writhing, and hind foot licking in mice [52]. Kirenol has dose-dependent anti-inflammatory effect equivalent to 1,2, and 4 mg/kg prednisone [14]. Ethanolic extracts were more effective when compared to water extracts in reducing joint swelling (P < 0.05) at doses of 4.8, 9.6, 16.384, 20.48, 25.6, 28 and 36 g/kg [53]. *S. pubescens* aqueous extract at concentrations of 50 mg/kg 25 mg/kg, and 12.5 mg/kg effectively suppressed PPAR-γ and NF-κB pathways in rats with ulcerative colitis. So ethanol significantly reduced inflammatory mediators in RAW264.7 cells and mice, ethanolic extract of Sigesbeckia *orientalis* 32 mg/kg BW/day reduced serum IL-6 and increased survival in vivo SOE also decreased ERK1/2, p38, and JNK phosphorylation, indicating anti-inflammatory effects [5, 54, 55]. SH reduced inflammatory cell proliferation by downregulating MyD88 and TRAF6 at aqueous extract doses of 4 g/kg, 2 g/kg, and 1 g/kg [56], and inhibited interleukin and TNF-α secretion at 20 mg/g in mice with odium urate-induced acute gouty arthritis [57]. SH aqueous extract dosage: 2.1, 4.2, 8.4 mg/kg Reduced IL-1β and TNF-α release and VCAM-1 expression in mice with Odium urate-induced acute gouty arthritis [58]. SG Methanolic extract 30 mg/kg decreases IL-3 and COX-2 production [6]. Administration of 500 mg/kg of SH A aqueous extract exhibited a reduction in serum TNF-α and IL-6 levels and ameliorated the kidney inflammation [59, 60]. SH extracts at 40 μmol/L target particular inflammatory mediators, with glabreside C, blocking the AKT/MAPKs pathway. This highlights the anti-inflammatory role of *ent*-pimarane diterpenoid dimers of SG. Glabreside C may be used to produce anti-inflammatory drugs [61]. Kirenol at doses of 10 μM, 20 μM, and 40 μM stimulates osteoblast development in MC3T3-E1 cells by activating the Wnt/β-catenin signalling pathway, leading to increased osteoprotegerin and osteopontin levels. Kirenol research may lead to new osteoporosis treatments [62]. *S. orientalis* at 2 g/kg protects cartilage in animal models of osteoarthritis by boosting sirt1 expression and lowering FOXO1 acetylation [63]. D101-adsorbed fraction of SO extract (purified active fraction derived from *S. orientalis* L.,) relates to perioperative neuroinflammation and neurodegenerative illnesses including Alzheimer's and multiple sclerosis, which are linked to microglial dysfunction and neuroinflammation, at 15–500 µg/mL [64]. Sigesbeckia species had lesser anti-inflammatory and toxicity effects than SO and SP in LPS-induced RAW 264 cells at 5–320 µg/mL. All species showed similar impact on the MAPK and NF-κB pathways, reducing LPS-induced COX-2 and iNOS levels during anti-inflammatory response [65]. Leocarpinolide B (LB) from *Sigesbeckia orientalis* reduced collagen type II-induced arthritis by inhibiting NF-κB DNA binding activity and interacting directly with NF-κB p65, indicating potential as an anti-rheumatoid arthritis treatment at concentrations of 10.0, 5.0, and 2.5 mg/kg [66].

### Anticancer

SG exhibits noteworthy anti-cancer effects, especially against MDA-MB-231 and MCF-7 breast cancer cell lines. The aqueous extract at a 1 mg/mL concentration induces cleavage of polymerase and ATPase, resulting in apoptosis in these cells. Furthermore, DKA at concentrations from SO (ethanolic extract, aerial parts) of 1, 5, and 25 μM impedes cell migration in wound healing assays and markedly decreases lung metastasis of MDA-MB-231 cells in murine models at dosages of 2.5, 5, and 10 mg/kg [67]. SO extracts, specifically those in ethyl acetate and *n-*butanol, demonstrate effectiveness against human lung cancer (A549) cells, cervical cancer (HeLa) cells, and endometrial (RL95-2) cells by inhibiting G2/M phase progression in vitro with IC_50_ 200 µg/mL, 50 µg/mL and 20 µg/mL respectively [68–70]. The sesquiterpenoids and terpenoids obtained from *S. orientalis* demonstrate IC_50_ values of 42 µg/mL, 5.1 µM, and 6.9 µM, against pancreatic cancer cell lines AsPC-1, PANC-1, and Hep-G2 [71]. SO inhibits tumour growth in vivo, decreases Bcl-2 gene and protein levels, suppresses tumour migration, and downregulates matrix metalloproteinases (MMPs) and β- catenin, IC _50_ value was 42 µg/mL [72].

### Anti-allergic

In vitro and in vivo evaluations of aqueous extracts from SP and SG *at dose* 1000 mg/kg, 500 mg/kg, 100 mg/kg, 50 mg/kg indicate their capacity to reduce B cell *IgE* production, implying a possible function in addressing allergies and immediate hypersensitivity [9]. The immunosuppressive activity of the ethanolic extract of *Sigesbeckia orientalis* (EESO) has been investigated, demonstrating that EESO at doses of 1.0 mg, 0.5 mg, 0.25 mg can inhibit both cellular and humoral responses to ovalbumin in mice. OVA-specific serum IgG2b, IgG1, and IgG levels were significantly decreased in OVA-immunized mice, suggesting its potential role as an immunosuppressant [73]. Furthermore, a delayed-type hypersensitivity (DTH) model induced by dinitrochlorobenzene (DNCB) demonstrated that SH extract at dose 15.0 g/kg, 7.50 g/kg, 3.75 g/kg can suppress the immunologic function in mice [74]. Kirenol at dose 2 mg/kg, a compound derived from SH, inhibits the proliferation of TH17 and TH1cells, reduces antibody generation, and diminishes T-cell-mediated delayed hypersensitivity, particularly affecting CD4^+^ T cells. Research indicates that hyaluronic acid may demonstrate antiallergic effects. Two hours post-administration of an oral dose of SP aqueous extract, cutaneous allergy symptoms exhibited a significant reduction [75]. The findings indicate the efficacy of various extracts and compounds from SH, underscoring their potential in developing therapies for allergies, immunosuppression, and related conditions.

### Anti-bacterial

The *methanolic* extract of (SG aerial parts) exhibits antibacterial effect, for instance the minimum inhibitory concentration (MIC) of a range 3.12 to 25 µg/mL against gram-positive bacteria. Kirenol exhibits a minimum inhibitory concentration MIC of 39 µg/mL against Bacillus *subtilis*, whereas its MIC for other bacterial strains varies from 78 to 625 µg/mL [7, 76]. The *(EE-SO) ethanolic* extract of SO demonstrates enhanced cytotoxic, antioxidant, and antimicrobial properties. Among the 12 isolated EE-SO compounds, several demonstrated significant antimicrobial activity, including 17,18-dihydroxy-ent-kauran-19-oic acid, 16β,17,18-trihydroxy-ent-kauran-19-oic acid, 16α,17-dihydroxy-ent-kauran-19-oic acid, and 16β-hydro-ent-kauran-17,19-dioic acid with MIC of 0.5 μg/mL and MBC = 1.0 μg/mL [77]. *S. orientalis Sh*ows a wider range of antimicrobial and biological activities, rendering it the more effective choice for potential therapeutic applications.

### Antioxidant

In 2014, researchers identified an extraction method utilising 80% ethanol (plant material to ethanol ratio of 1:10 at 60 °C for two hours) as the most effective for free radical scavenging, anti-inflammatory, and anti-lipid peroxidation activities. The SH extract (170 and 340 mg/kg) enhanced catalase (CAT), superoxide dismutase (SOD), and free radical scavenging in living organisms for seven days [78]. In the model group, there was a significantly higher levels of malondialdehyde (MDA), and reactive oxygen species (ROS) in synovial cells, followed by a marked reduction of superoxide dismutase (SOD) levels. SH enhances Nrf2 synthesis and its nuclear translocation by modulating synovial cells via the Nrf2/HO-1 signalling pathway, thereby promoting HO^−1^ expression and mitigating oxidative stress responses, doses used were 4 g/kg, 2 g/kg, 1 g/kg [58]. *n*-butanol fraction of SH increases cellular antioxidant capacity by activating defence genes, including G6PDH, GCL-m, and GCL-c, which reduces oxidative stress. SH extract 200 μg mL^−1^ combats against PM_10_ toxicity explained by the chlorogenic acid that enhance the antioxidant capacity of the cells. It is the protective activity of the SH extract at 200 μg/mL against PM10-induced toxicity that is attributed to chlorogenic acid as an active component. [7]. *S. orientalis* exhibits the broadest spectrum of beneficial activities among the extracts analysed, notably in its antimicrobial and antioxidant properties, positioning it as a viable candidate for therapeutic use.

### Antithrombotic

In a rat blood stasis model, the 70% ethanol extract of SP: 110 mg/kg, 55 mg/kg demonstrated a reduction in plasma fibrinogen levels and inhibited endothermal release. DDKA: 200—1000 μg/mL demonstrates significant anticoagulant and antithrombotic properties, reducing AVB thrombus weight, elevating plasma cAMP levels, extending thrombin time, and inhibiting platelet aggregation. Thrombosis is defined by the coagulation of blood and the development of solid aggregates composed of insoluble fibrin. The methanol extract of Sigesbeckia glabrescens (MESG) induced endothelium-independent vasorelaxation in isolated rat thoracic aorta rings, suggesting direct smooth muscle relaxation without involvement of endothelial nitric oxide pathways [11, 79].

Kirenol: 1000 mg/kg, 500 mg/kg, 250 mg/kg is likely the most effective anti-thrombotic component in SH, with diterpenoids recognized as the main compounds in the antithrombotic fraction of SH [80]. The aqueous extract of SH reduces platelet aggregation rates, haematocrit values, and the viscosity of plasma and whole blood, while it accelerates the sedimentation rates of erythrocytes and prolongs prothrombin and thrombin times [47]. Preclinical studies suggest that Xi-xian-Cao (Herba Sigesbeckiae), particularly its n-butanolic extract at 50 μg/mL, may offer neuroprotective and anti-thrombotic benefits in ischemic stroke models. However, its clinical relevance remains to be validated through rigorously designed human trials [8]. SP demonstrates a wider array of beneficial effects in thrombotic conditions, making it a promising candidate for further therapeutic investigation.

### Cerebral ischemia protection

Supplementation of SH at doses of 400 and 800 mg/kg resulted in decreased cerebral vascular permeability and reduced brain water content, indicating its potential efficacy in treating cerebral ischemia. Severe transient cerebral ischemia results in neurological system damage, leading to limb numbness and weakness [14]. The extract of SH has been shown to decrease cerebral infarction volume, enhance neurological function recovery, inhibit nerve cell apoptosis and inflammatory factor expression, and promote NeuN neuronal antigen expression, thus providing protective effects in MCAO rats. SH may confer neuroprotection against ischemia–reperfusion injury by enhancing anti-oxidase activity, improving cerebral blood flow, and mitigating blood–brain barrier disruption [75]. SH improved G*SH*-Px and SOD action, decreased MDA levels, and mitigated blood–brain barrier disruption in mice experiencing cerebral ischemia. The impact of SH on cerebral ischemia has attracted attention recently. 5,3′-dihydroxy-3,7,4′-trimethoxyflavone enhances the protection of nerve cells through the upregulation of Cyclin dependent kinase (CDK5), Mitogen- activated protein Kinase 10 (MAPK10), and Mouse Double Minute 2 homolog (MDM2). Activation of FLT1 (Fms-Related Tyrosine Kinase 1) and FLT2 (Fms-Related Tyrosine Kinase 3) vascular endothelial growth factor receptors facilitated vascular repair and regeneration. SH mitigates cerebral ischemia, as indicated by TCM [81]. SH may mitigate ischemia–reperfusion injury in the brain by enhancing antioxidant activity, improving cerebral blood flow, and reducing blood–brain barrier damage.

### Cardio protection

Research indicates that SH exhibits an anti-hypertensive effect. The study concluded that SH affected endothelial NO-dependent vasodilation as demonstrated by the rat thoracic aorta rings method. Not only that, but SP does not directly relax vascular smooth muscle; instead, it stops sympathetic vasoconstrictor nerves from working.,3′ -dihydroxy-3,7,4′—trimethoxyflavone: at doses of 40 μM, 20 μM, 10 μM, 5 μM, 1 μM has neuroprotective impact via the signalling pathways regulation of brain-derived neurotrophic factor and P13k-Akt, for example upregulating the expression of MDM2, MAPK10, and CDK5 [82]. A high dose of SH extract mitigates myocardial remodelling in rats subjected to pressure overload [83]. SH *aqueous extract:* 12 mg/mL, 10 mg/mL, 8 mg/mL, 6 mg/mL, 4 mg/mL, 2 mg/mL reduces acute cardiac injury caused by DOX in rats, potentially due to its antioxidative properties [84]. SH *ethanolic* extract: 9.6 g/kg, 4.8 g/kg reduced the animal model systolic and diastolic blood pressure and improved hemodynamic and heart weight index [85]. SH *ethanolic* extract: 340 mg/kg protected the doxorubicin-induced myocardial injury [86]. Kirenol: 40 μmol/L, 20 μmol/Lhas important heart-protecting effects in the treatment of established diabetic cardiomyopathy. It does this by blocking signalling pathways related to inflammation and fibrosis, regardless of how it affects hyperglycaemia, hyperinsulinemia, or lipid profiles [87].

### Antidiabetic

*Ent*−16, 17-isobutyryloxy-kauran-19-oic acid non-competitively inhibited PTP1B, reducing blood glucose levels. The aqueous and *ethanolic* extracts of SO (500 mg/kg) enhanced insulin sensitivity in mice with streptozotocin-induced diabetes. Aqueous extract demonstrated superior efficacy compared to *ethanolic* extract of SO [88].

### Uricosuric agents

SH extract at concentrations of 2.1, 4.2 and 8.4 g/kg demonstrated superior reductions in creatinine, urea nitrogen, and uric acid compared to allopurinol with IC_50_ = 8.7 ± 0.9 μg/mL [89].

### Fibroblast stimulator

In rat thoracic rings, SH induced endothelial nitric oxide-dependent vasodilation. Excision-injured rat fibroblasts were stimulated by SO extract at concentrations ranging from 20 to 200 g/mL. DHK facilitates Akt-mediated phosphorylation of the Y1068 growth factor receptor, thereby enhancing keratinocyte stem cells proliferation and migration. SH improves skin wound healing [14, 90, 91].

### Cosmetic: skin whitening

SGMFAb, derived from SG, demonstrates potential as a skin-whitening agent through the inhibition of melanogenesis and related melanosome transport processes in B16BL6 cells. The expression of melanogenic proteins and MAPKs in these cells is reduced, leading to a decrease in α-M*SH*-induced elevations of melanosome transport proteins. SO, a plant utilized for skin lightening in traditional practices. Investigating its efficacy as a bleaching agent and its retinoid-like properties may uncover parallels with B. *pilosa*, known to be originating from Asteraceae family. Flavonoids are likely responsible for the whitening properties of SO, indicating its potential as a formulation for whitening, anti-blemish, and anti-aging applications [19, 48].

All three species demonstrate notable therapeutic potentials; however, SO is distinguished by its extensive antimicrobial, antioxidant, and cytotoxic activities, positioning it as a leading candidate for further research and development in therapeutic applications. SG is recognized for its anticancer properties and potential applications in osteoporosis treatment, whereas SP is distinguished by its anticoagulant effects and neuroprotective capabilities. SO warrants further investigation, especially regarding its potential in treating diverse cancers and inflammatory conditions, while SP *Sh*ould also be considered for its cardiovascular and immunological advantages. SG is significant, particularly in the fields of oncology and bone health.

### Secondary metabolites: update

As of 2024 and solely from SG, SP, and SO, 310 compounds were identified and their chemical structures were characterized comprising secondary metabolites such as flavonoids, diterpenoids, sesquiterpenoids, triterpenoids, sterols, oxylipins, and other small organic compounds. The compounds IUPAC names are presented and summarised in the Supplementary Table after the document.

### Flavonoids

**Fig. 12:**
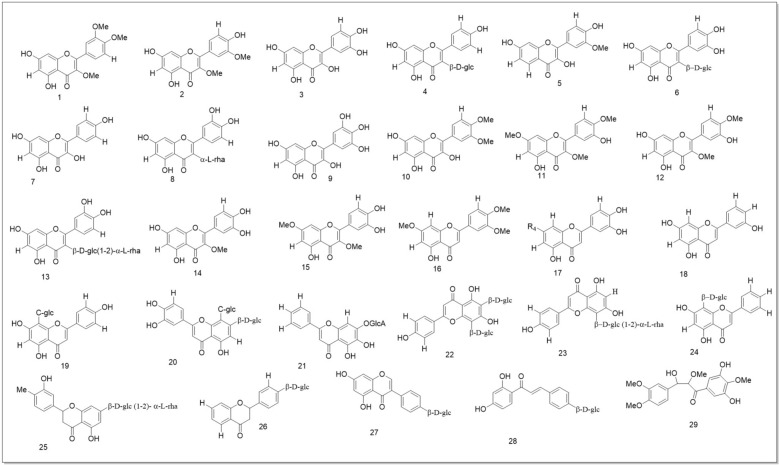
Structures of flavonoids identified from SH

Figure 12 shows the structures of 29 SH flavonoids. Rutin **(13)** and 3-methylquercetin **(14)** were specifically discovered in SO/SP and SP/SG, respectively, whereas 3,7-dimethylquercetin **(15)** was found in all SH species. Two flavonols, 3,7,4′-O-trimethyl quercetin **(11)** and 3,4′-O-dimethyl quercetin **(12)**, were found in SG [69, 91–96]. A detailed examination of SP found 15 flavonoids: 10 flavonols **(1–10)**, 9 flavones **(16–24)**, 2 dihydroflavones **(25–26)**, 1 isoflavone **(27),** and 2 chalcone derivatives **(28–29)** [95, 97, 98]. Importantly, 24 of the 29 flavonoids are flavonols compounds **(1–15)** or flavones compounds **(16–24),** showing that SH species produce most of their flavonoids in these two categories.

The distribution of flavonoids among species is unclear since most research focuses on SP and less on SO and SG. More study is needed to determine if SH plants or species have unique flavonoids, as these compounds are found in many plants.

### Terpenoids

**Fig. 13:**
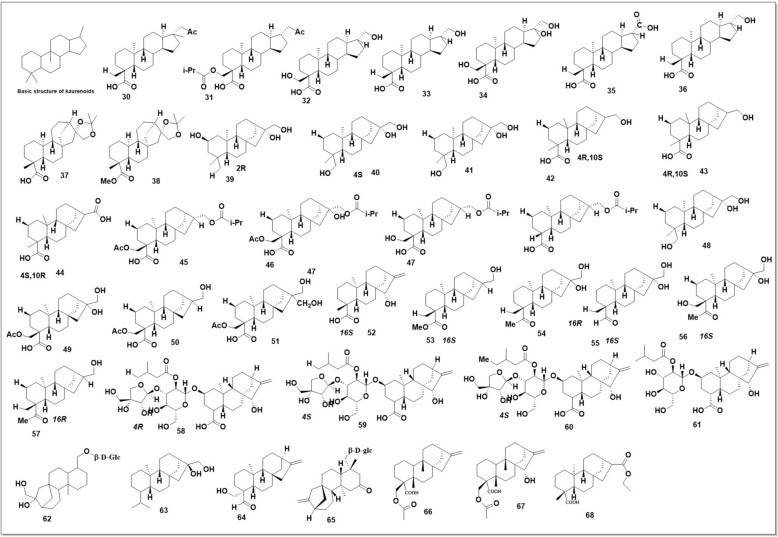
Kaurenoids present in SH reported to date

Diterpenoids and triterpenoids have been identified from SH. These are some of the most important types of chemicals in SH herbs. The representative chemical skeletons of SH diterpenoids are tetracyclic kaurane and tricyclic Pimarane as shown in (Fig. 13). Sesquiterpenoids have also been known from SH.

### Kaurenoids

Kaurane-type diterpenoids (KDPs) have a perhydrophenanthrene moiety (A, B, and C rings) joined with a cyclopentane D ring. C-8 and C-13 are joined by a carbon bridge **(**Fig. 13**)** [99]. Kaurane diterpenoids have C-16, C-17, C-18, and C-19 substituents, with some having C-2 replacements. Figure 13 shows the chemical structures of three SH species' KDPs. Four KDPs with carboxyl groups at C-19 were identified from SO aerial parts: siegesetheric acid I **(30)**, siegesesteric acid II (31), annosquamosin A (32), and ent-16β,17-dihydroxykauran-19-oic acid-16β,17-acetonide **(37)**, respectively [94, 100]. SP and SG reported compounds **(30)** and **(37)** [94, 100]and compound **(32)** [41, 42, 100, 101]. Three SH KDPs **(39–41)** without a carboxyl moiety at C-19 were previously discovered. Trihydroxy groups at C-2, C-16, and C-17 on its kaurane-type diterpenoid carbon structure changed Siegesbeckiol **(39)** [102]. *Ent*-kauran-16β,17,18-triol **(40)** and kaurane-16,17,18-triol **(41)** are stereoisomers. Most KDPs from SP have a carboxyl moiety at C-19, like those from SO. Substituted esterification at C-19 distinguishes compound **(38)** from compound **(37)** [41, 42].

Three further kaurane-type diterpenoids from SP are structurally similar to compound 40: ent-17,18-dihydroxy-16βH-kauran-19-oic acid **(33)** [39, 103, 104], ent-16α, 17,18-trihydroxykauran-19-oic acid **(34)** [41, 42, 105], and siegeskaurolic acid **(36)** [106]. Siegeskaurolic acid's C-17 carboxyl group was modified by compound **(35)** and its stereoisomer, kauralexin A2 **(42)**. Three further stereoisomers of siegeskaurolic acid were found in SP: (-)−17-hydroxy-16α-kauran-19-oic acid **(43)** [41, 42], ent-17-hydroxy-16βH-kauran-19-oic acid **(44)**, and ent-16α, 17-dihydroxy-kauran-19-oic acid **(55)** [100, Compounds **(45–48)** have C-19 carboxyl and C-17 isobutyrate groups. Compounds **(49–51)** have C-19 carboxyl and C-18 acetic acid ester [41, 42, 107]. Compound **(51)** has a C-17 carboxyl group, unlike **(49)** and **(50)**. Grandifloric acid **(52)** has a C-16–C-17 carbon–carbon double bond [39]. A methyl ester replaces the carboxyl group at C-19 of compounds **(53–54)** and **(56–57)**. Stereoisomers **(53)** and **(57)** were found. Four *ent*-kaurane glucopyranosides **(58–61)** were also isolated from SP aerial parts [107]. The *ent*-kaurane glucopyranoside seigesbeckioside **(62)** was previously isolated. Siegeside E (65), a new SP glycoside, was discovered [39, 108]. SG also has compounds (**36, 48, 56**, and **62**) [101, 109, 110]. SG contains other kaurane diterpenoids such as 16β,17-dihydroxy-kaurane **(63)** and (4β)−19-hydroxy-kaur-16-*en*−18-oic acid **(64)** [102]. Sigesbeckia orientalis yielded eleven compounds, including three novel *ent*-kaurane diterpenes: 18-O-acetyl-kauran-16-ent-19-oic acid, sigesbeckin B, and sigesbeckin C **(66–68)**. The unique chemicals, not previously documented in SH reviews, help create antibiotic alternatives. Significant progress in combating antibiotic resistance is shown by a minimum inhibitory concentration (MIC) of 64 μg/mL [111]. Since **(31)** of 39 KDPs were found in SP, KDP may be a group of SH compounds. Few KDPs in SO and SG may be due to poor study. KDP may also represent SP's compounds to distinguish it from other SH species. Although both concepts are promising, further study is needed to validate them, indicating future potential in that subject.

### Pimarane-type diterpenoids

**Fig. 14:**
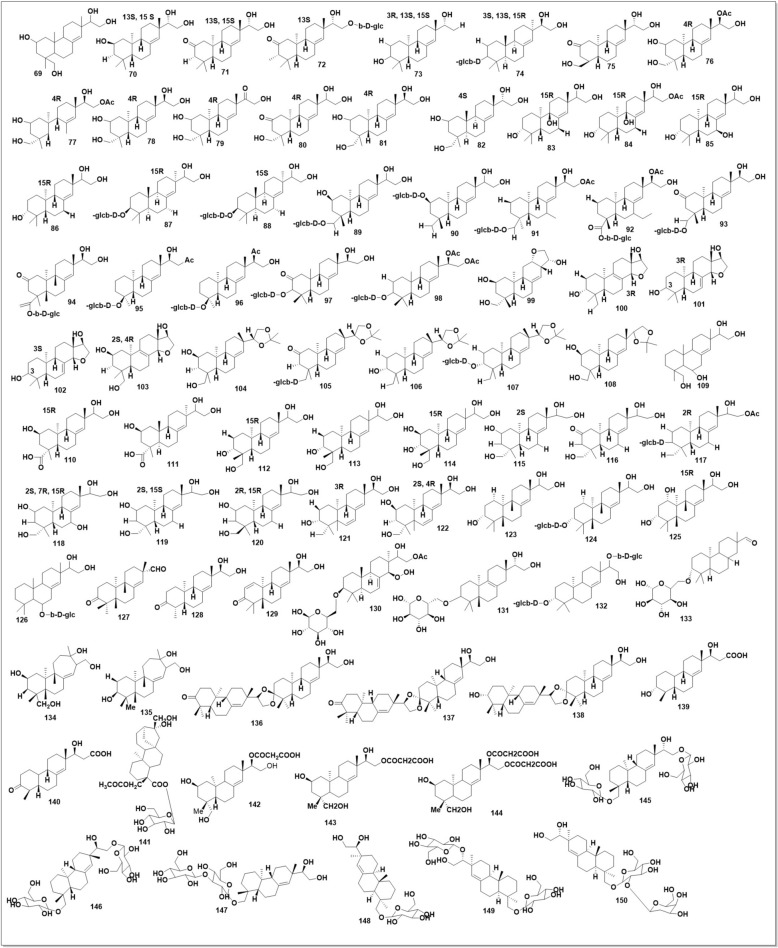
Structure of *ent*-pimrane found in SH to date

A perhydrophenantrene unit (A, B, and C rings) with an angle methyl at the C-4 position of the A ring and methyl ethyl at the C-13 position of the C ring makes up the marine-type diterpenoids (PDPs). Substitutions mostly occur at C-2, C-3, C-15, C-16, C-18, and C-19. C-6, C-7, and C-14 may be substituted. Additional structural distinctions include perhydrophenantrene's carbon–carbon double bond locations. (Fig. 14**)** shows the chemical structures of all plant PDPs, revealing their makeup.

C-8 and C-14 produced a carbon–carbon double bond in most SO PDPs **(69–72, 75–80, 83–87, 89–99, 104)**. C-2 substitutions divide compounds **(69–80)** into hydroxyl and glycosylation groups. Compounds **(71–73** and **79)** have a carbonyl group at C-2, whereas 72 and 80 are stereoisomers [112, 113]. Compound **(116)** is a stereoisomer of SP compounds **(75)** and **(80)** [107]. Another PDP cluster with a C-2 hydroxyl was named: Ent-2α,15,16,19-tetrahydroxypimar-8(14)-ene **(69)** [113], 2β,15,16-trihydroxy-ent-pimar-8(14)-ene **(70)** [112], orientalin A **(76)** [94], B **(77)** [113], kirenol **(78)** [41, 42, 113], and ent-15-oxo-2β,16,19-trihydroxypimar-8(14)-ene, compound **(115)** is a stereoisomer of SP compounds **(69)** and **(78)** [107]. Three compounds (83–85) have C-3 hydroxyl substitutions. Compounds **(83)** and (84) were hydroxylated at C-9, whereas **(80)** was at C-7. Compound **(123**), a stereoisomer from SP [107], yielded darutigenol **(86)** [41, 42]. Five ent-pimarane glucopyranosides with a glycosidic link at C-3 were recovered from SO: darutoside **(87)**, hythiemoside A (95), 15,16-di-O-acetyldarutoside **(98)** [41, 42], hythiemoside B **(96)** [38], and *ent*−2-oxo-3β,15,16-trihydroxy-pimar-8(14)-en-3-O-β-glucopyrano. *Ent*-pimarane glucopyranosides **(117 and 124)** from SP are stereoisomers of hythiemoside A and darutoside [107]. Pimarane diterpenoids often have an ethylene glycol molecule in the side chain at C-13–16, as in six more *ent*-pimarane glucopyranosides **(89–94)** [113]. The replaced glycoside was in a different position. Pubeside A **(90)** formed a glycosidic bond at C-2, whereas the others formed at C-18's side chain. Pubeside D (93) and compound (94) produced carbonyls at C-2. Pubeside C **(92)** and compound 94 carbonylated their glycosidic carbons. Oxolane was formed between compound (99)'s C-12 and C-16 by an oxygen bridge [113]. Different **compounds (100 & 103)** created the oxygen bridge between C-14 and C-16 [41, 42]. The carbon–carbon double bond in these compounds was formed between C-8 and C-9. Isopropylidenekirenol **(104)** [113] had a carbon–carbon double bond between C-8 and C-14 and a five-membered dioxygen heterocyclic moiety at C-15–16. Orientalin B **(77)** [40, 69, 113], kirenol **(78)** [40–42, 113], darutigenol **(86)** [38, 41, 42, 107], darutoside **(87)** [41, 42, 107], and isopropylidenekirenol **(104)** [40–42, 113] were also found in SP.

Four further SP-derived PDPs (105–108) have a Shared dioxo-5-membered ring moiety at the C-15–16 side chain [41, 42, 107, 113]. Compound (108) is isopropylidene kirenol's stereoisomer. Compound (109) was replaced by a hydroxyl at C-6 of compound **(69)** [114]. Stereoisomers **(110)** and **(111)** are separated from SP. Both stereoisomers **(112)** [41, 42] and **(116)** [107] are separated from SP. The C-6 glycoside substitution in compound (129) was unique [115]. Carbonylation and aldehyde substitution occurred at compound **(127)**'s C-3 and C-13–16 side chains [116]. Compound (130) produced a carbon–carbon double bond between C-7 and C-8, unlike previous SP PDPs. However, the hydrogen peroxide substitution at C-14 of compound **(130)** may be unstable, causing an allylic rearrangement and a delayed Smith epimerization to a different hydroperoxide [41, 42]. Siegeside B aglycon **(73)**, siegeside A **(74)**, compound **(81–82)**, siegeside B **(88),** and compound **(101–102, 114, 118–120, & 125)** were isolated from SP recently [117]. Two carbon–carbon double bonds were found in SP compounds **(121–122)**. One was C-6 and C-7, the other C-8 and C-14. Two diterpene glycosides were found in SP. Siegeside D (133) has an aldehyde group at C-15, whereas siegeside C **(132)** has a carbon–carbon double bond between C-8 and C-9 [117]. Neodarutoside **(131)**, with two glucopyranoside subunits at C-3 and C-15, was isolated from SG [107]. The pimarane diterpenoid obtained from SO was first identified as ent-12α,16-epoxy-2β,15α,19-trihydroxypimar-8-ene [113], but later renamed as compound **(103)** [41, 42]. Darutin was isolated from uncharacterized SO. Two *ent*-strobane diterpenoids, strobols A (134) and B (135), were recently identified from SP [108].

SG flavoside A-C **(136–138)**. Glabreside C increased HO-1 protein expression in LPS-stimulated BV2 cells while suppressing iNOS and COX2. Glabreside C inhibits AKT/MAPK signalling to reduce inflammation. *Ent*-pimarane diterpenoid dimers have a key part in SG’s anti-inflammatory effect, providing crucial proof for SH’s development and use. Glabreside C may be a viable starting chemical for anti-inflammatory drugs [118]. The discovery of Sigesbeckia K and L, new glabrescens diterpenoids, as potential anti-inflammatory agents is a significant finding. These substances, tested for their anti-inflammatory properties by inhibiting LPS-induced NO generation in BV2 microglial cells, showed promising inhibitory effects, with an IC_50_ of 62.56 μM, compared to the positive control minocycline at 32.84 μM. This discovery paves the way for further research into these compounds and their potential as anti-inflammatory agents [116]. The thorough examination of S. pubescens from Primorsky Territory, which led to the isolation of the diterpenoids *β*-glucopyranosyl-18-acetoxy-16*α*, 17 dihydroxykauran-19-oate **(142)**, 15-*O*-malonylkirenol **(141)**, and 16-O-malonylkirenol **(143)**, provides a solid foundation for the research. The structures of the new diterpenoids **(141–143)** were established by NMR and mass spectrometry, ensuring the reliability of the findings [117]. Three SH species yielded 48 chemicals. SG yielded 15,16-di-*O*-malonylkirenol **(144)**. The structure was revealed by IR, HR-ESI–MS, and 1-D and 2-D NMR, including 1H, 13 C, DEPT, 1H-1H COSY, HMQC, and HMBC spectra. The other chemicals were identified using mass spectral data and earlier investigations [119]. SG aerial parts yielded six new diterpenoid glucosides, siegeside Q–V **(145–150)**, and four recognized chemicals. SG glycosides may have anti-inflammatory effects, supporting their medicinal use. Compound 1's diterpenoid moiety has 4R, 5S, 9R, 10S, 13S, and 15S stereochemistry [120]. PDPs have been found in total is 82 from SH species, suggesting they may be another important category of chemicals. All three species share 35 PDPs. Only 13 are in SG, 2 in SO, and 31 in SP. Insignificant structural difference between SO and SP, PDP distributions. Comparative studies are needed to determine species-specific PDP content. The chemical makeup of SG and SO is less studied than SP, requiring additional study.

### Sesquiterpenoids

**Fig. 15:**
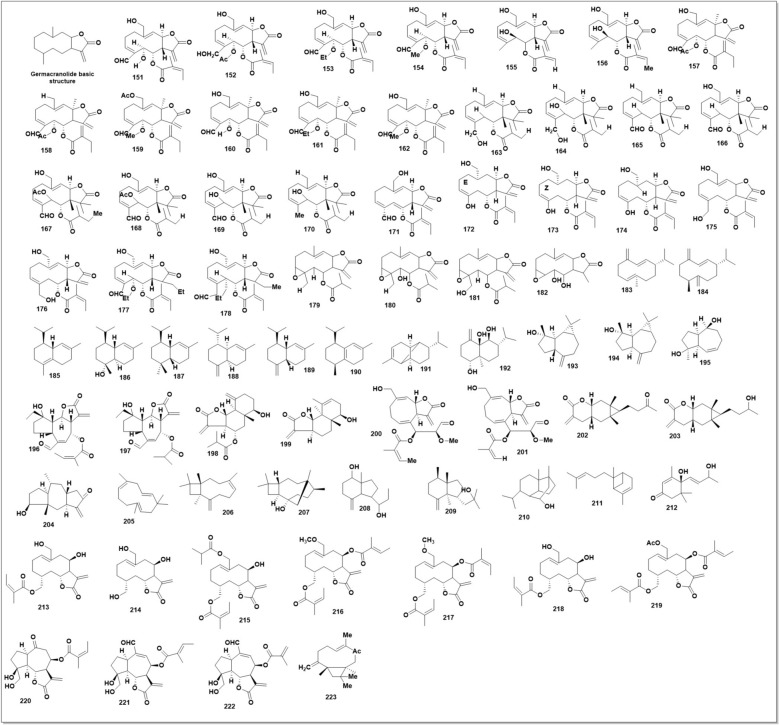
Sesquiterpenoids identified from SH

The number of sesquiterpenoids reported in SO, SP and SG were 35, 26 and 14, respectively. These sesquiterpenoids could be classified into germacranolides, cadinane and other types. All the chemical structures of sesquiterpenoids found in SH plants are shown in Fig. 15.

Compounds (**151–182**) shared a similar carbon skeleton constituted of a germacrane ring fused with a heterocyclic, formed by* γ*-lactonization between C-8 and C-12. Also, a carbon–carbon double bond between C-11 and C-13 was in common (Fig. 15). Possible substitutions would occur at C-5, C-6, C-14, and C-15. And the position of carbon–carbon double bond within the germacrane ring would also be differentiated. Among them, compounds **(151–159, 163–172, 175–182**) were isolated from SO [112, 121, 122]. Compound (**160- 161**) were isolated from SP [107] while compound (**173–174**) [123, 124] were isolated from SG. A germacranolid named as pubetallin (**160**) was found in both SP and SG [121, 125]. Uniquely, an oxirane moiety between C-3 and C-4 was formed among compounds (179- 182) [112]. (Compounds (**183- 184**) were two other germacranolides identified in the leaf oil of SP, which without heterocyclic subunit in structure [126].

Series of cadinane sesquiterpenoids (**185–190**) were found only in SP, distingui*sh*ed by different number and location of carbon–carbon double bonds. Compound (**185- 188**) were identified in leaf oil of SP [126], while compound (**189–190**) was identified from volatile oil in the stem and leaf of SP [127]. Moreover, compound (185, 186) and compound (187, 189) were two pairs of stereoisomers, respectively.

Two eudesmane sesquiterpenoids, namely α-copaene (191) and 1β,6α-dihydroxy-4(14)-eudesmene (**192**) were found in SP [126]. Spathulenol (193) and (+)−4a-allospathulenol (**192**) were two aromadendrane sesquiterpenoids also found in SP [126, 127]. Three guaianolides compound **(195–197)** were isolated from SO [122]. Earlier, two eudesmanolides compound (198–199) were also isolated from SO [112]. In addition, two sesquiterpene lactones, namely as siegenolide A (**200**) and siegenolide B (201), were isolated from SG together with compound (**173–174)** [123]. Three other sesquiterpene lactone compounds (**202- 203**) were isolated from SP [107]. Other sesquiterpenoids, compound (**204–212**) found in SP [107].

*β*-​Caryophyllene oxide has been identified from SO (**213**). As sesquiterpenoids found in SH plants are common compounds, it might not be a representative group of compounds for SH. However, as germacranolides mainly occur in SO, while cadinane sesquiterpenoids mainly occur in SP, it suggests that sesquiterpenoids might be suitable chemical compositions to distinguish different SH species [15].

Chromatographic fractionation of SG led to the identification of 10 new sesquiterpene lactones (**214–223**), named siegesbeckialides I-O (1–7) (**214–221**) and glabrescones A-C (**222–223**). Mechanistically, siegesbeckialide-I directly binds to inhibitors of IKKα/β and suppresses their phosphorylation. This leads to the inhibition of IKKα/β-mediated phosphorylation and degradation of inhibitor α of NF-κB (IκBα), as well as the activation of NF-κB signalling [61].

As sesquiterpenoids found in SH plants are common compounds, they might not be a representative group of compounds for SH. However, as germacranolides mainly occur in SO, while cadinane sesquiterpenoids mainly occur in SP, it suggests that sesquiterpenoids might be suitable chemical compositions to distinguish different SH species.

### Lignoids from Herba *Sigesbeckiae*

**Fig. 16:**
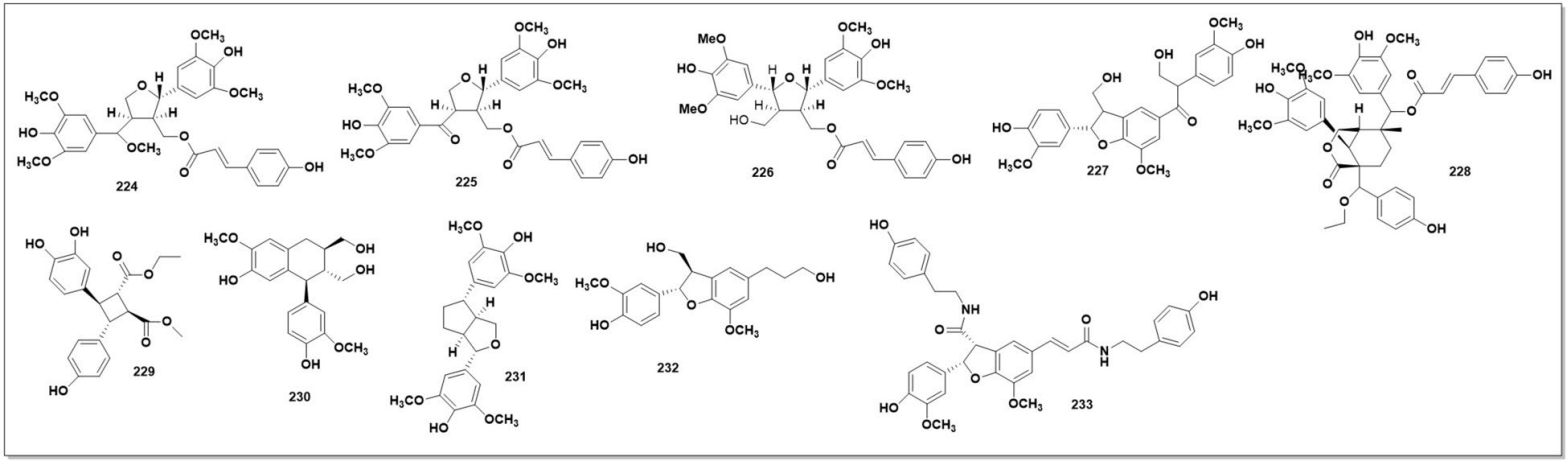
Lignoids identified from SH

Six novel lignanoids, Glalignin A-E (**224–230**) and Glaneolignin A, along with four analogues, (+)-isolariciresinol (**231**), (+)-syringaresinol (**232**), dihydrodehydrodiconiferyl alcohol (**233**), and tribulusamide A **(233),** were isolated from the SG (Fig. 16). The structures of these compounds were determined via HRESIMS, 1D and 2D NMR, and chemical evidence. The MTT assay measured how many tumour cells the chemicals inhibited. Novel compounds (**226** & **230)** had IC_50_ values of 32.89 6.83 M and 35.86 6.83 M, respectively, for human lung adenocarcinoma cells A549 [128].

### Steroids and oxylipins

Three ursane-type pentacyclic triterpenoids (**234–236**) [97] and six steroids (**237–244**) [39, 61, 97] have been isolated from SP, including β-sitosterol (235) and β-daucosterol (236), which have also been found in SO and SG [100, 102]. Other aliphatic organic acids found in SH species have been shown in (Fig. 19)*.* These compounds are quite common among different plants, which might not be meaningful for the quality control of SH. However, their bioactivities Should not be ignored. Three oxylipin (**245–247**) have been identified from SG [129]. **Seven** rare oxylipins were separated and identified from the aerial parts of SG *in* 2021 [130]***. Sigesbeckin A-C* (248–254).** Ten new phyto-oxylipins siegesoxylipin A‒J (**255–264**), these were isolated from the aerial parts of SO [131]. All the mentioned secondary metabolites in Fig. 12, 13, 14, 15, 16, 17 are mentioned in supplementary Table S2. Rest of the compounds are aliphatic acids, phenolic acids carbamate mentioned in supplementary Table S3.

**Fig. 17:**
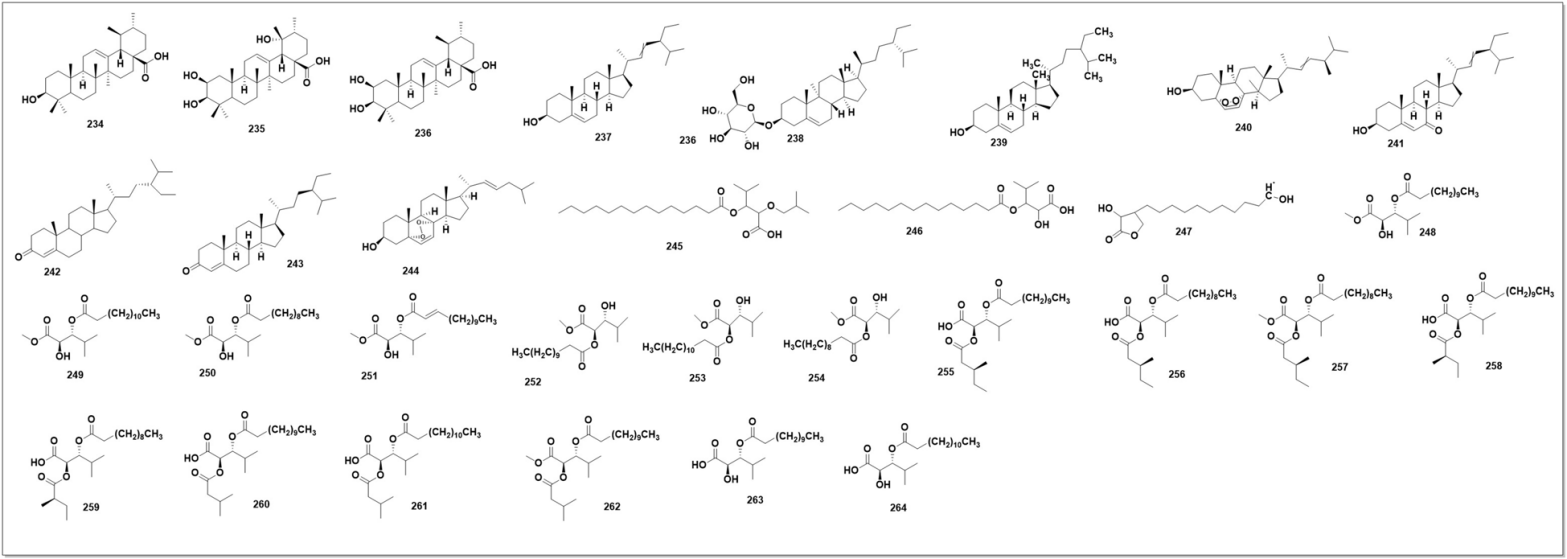
Steroids and oxylipins known to date from SH

## Conclusion

Thy food is thy medicine," said Hippocrates. The *Sigesbeckia L*. genus is a significant source of important secondary metabolites. SH species are rich in bioactive components, nutrients, and medicinal properties that benefit good health. The current study focuses on the research on various features of solely three *Sigesbeckia* species: Sigesbeckia *glabrescens* Makino, Sigesbeckia *pubescens* Makino, and Sigesbeckia *orientalis L.* The additional species reported in the literature that belong to the genus *Sigesbeckia L*. are also mentioned in this work and have still to be investigated. A total of 308 compounds were extracted from SO, SP, and SG, including 29 flavonoids, 39 kaurane diterpinoids, 82 pimrane diterpenoid, 73 sesquiterpinoid, 10 lignoids, 31 steroids, and oxylipin. The remaining compounds are aliphatic and cyclic hydrocarbons. Several of these elements have been investigated for diverse biomedical functions. For instance, 2β-Hydroxyursolic acid, β-Sitosterol, Sigesbeckin A-E, quercetin-7-*O*-β-d-glucoside, Siegeside Q, siegesbeckialides, Sigesbeckia K and L, 15-*O*-malonylkirenol, 16-*O*-malonylkirenol, glabreside* C*, isorhamnetin, quercetin, and kirenol. Kirenol, present in all three species of SH, suppressed TNF-*α* induced cell viability, migration, inflammatory cytokine production, and anti-oxidatent impact in HaCaT cells by reducing MDA levels and decreasing the NF-κB signaling pathway [132]. As a result, it might be an effective treatment for psoriasis and other skin conditions. Most of the pharmacological activity were investigated on SP, whereas SO and SG were assessed for antioxidant, antibacterial, antipyretic, and anti-inflammatory properties. The leaves are the most often utilized portion in all three species. This review study finds that SH extracts have strong antidiabetic, adaptogenic, antiarthritic, anti-inflammatory, and antibacterial properties in vitro and in vivo. However, employing various model systems, there is a call to determine the mechanism of action beyond their cardioprotective, antibacterial, antioxidant, and anticancer effects. SH extracts displayed a broad spectrum of biological actions in vivo including antimicrobial, antipyretic, anti-inflammatory, and antirheumetic, among others, with concentrations ranging from 4 mg/kg to 500 mg/kg, indicating that 4 mg/kg is the lowest concentration and most potent. In comparison, 500 mg/kg is not toxic. In 2012, Han et al. discovered that S. orientalis may be administered at up to 14 g/kg for ischemia reperfusion without adverse effects. The different secondary metabolites such as flavonoids, kauroids, pimarane, sesquiterpinoids, lignoids, oxylipin, or the complete plant of Sigesbeckia species have demonstrated a synergistic impact behind all pharmaceutical activity. These findings lend credence to the genus *Sigesbeckia L.*'s traditional usage in treating various maladies, such as fever, cold, malaria, diabetes, inflammation, liver, and heart diseases. Although various chemicals from SP, SO, and SG have been identified, their biological potential has yet to be investigated. Thus, the paper demonstrates that S. glabrescens is the least investigated species especially for pure extraction of chemicals. However, current papers show that interest in SG has risen. Clinical testing of crude extracts and purified chemicals from the lesser-known SH species in vivo is still necessary. Furthermore, the molecular action mechanism of the extracts and other components of the plants need to be thoroughly investigated in vivo. This work aims to assist researchers recognize the medicinal impacts of SH. Few limitations are associated with this work. First, the articles were obtained from a single database since VOS Viewer's data structure needed consistency. It may be enhanced by combining data from Web of Science, Google Scholar, and Scopus to generate the complete results. Furthermore, the Scientometric findings are summarized based on the authors' expertise, which may limit its comprehensiveness. In the future, publications can be divided into groups, and the results of the Scientometric analysis in each group will be submitted to the corresponding expert, where experts from various fields can further present research gaps and findings to increase the comprehensiveness of suggestions.

In the future, the Scientometric results will be sent to experts in the same area of interest, sheding the light on the research gaps, possibilities, and limitations. The increased number of papers in this subject demonstrates that SH is becoming more widely accepted as a viable treatment for disorders. China has taken the lead in working on SH, with Korea and the United States following closely. However, the regional spread shows the breadth and diversity of studies on this plant. Moreover, the noticed lack of inter-organizational and inter country cooperation can demonstrate the need for collaborative initiatives to boost this front in the field of traditional and modern medicine based on plants. Keyword repeated occurrence highlights the interest growth, demonstrating a shift toward investigating the confluence of SH with emerging directions, including rheumatic arthritis, dermatitis, and other skin illnesses. This multidisciplinary approach, incorporates nanotechnology, pharmacology, and immunology, and shows the flexibility and promise of TCM and herbal medicine research. Although the number of publications on Sigesbeckia herba (SH) remains relatively limited, this does not necessarily indicate a lack of scientific value. In fact, the available literature suggests that the three Sigesbeckia species have been investigated in considerable depth from a phytochemical perspective. Continued research into SH and related species can further enrich our understanding of their pharmacological potential and support the development of plant-based therapeutics. This area of research carries the promise, hence demanding joint efforts to explore future directions. Citation analysis revealed the most significant scholars in the area, giving a road map for future researchers and emphasizing the importance of contributions from a broader range of geographies and disciplines. In 2015, the **UK's Medicines** and Healthcare Products Regulatory Agency (MHRA) approved Phynova Joint and Muscle Relief Tablets™, a traditional herbal treatment derived from SH aerial components. This support was owned to scientific evidence demonstrating the SH use for over three decades, with at least half of those years happening within an EU member state. This is derived from Sigesbeckia pubescens (British Herbal Medicine Association). These findings will enhance collaboration and fair research possibilities in the TCM/herbal medicine sector. Herbs have the treatment for a wide range of disorders. We encourage funders and policymakers to recognize and resolve regional TCM and herbal medicine research inequities. The use of nature, specifically herbs and plant sources, can leverage collaborative efforts toward improving various therapies, particularly arthritis, whether it is rheumatism, psoriatic arthritis, or gout, strengthen the research foundation and enhance the patients’ medical conditions globally. According to Avicenna, "there are no incurable diseases, only a lack of will." There are no useless herbs, only a lack of understanding. "Research on herbal medicine, and Traditional Chinese Medicine has been promising, but its full potential has yet to be realized. Many uncharted avenues and unresolved questions exist, and they may be pursued with interest teamwork, and defiance.

## Supplementary Information

Supplementary file 1

Supplementary file 2

Supplementary file 3

## Data Availability

The datasets used and/or analyzed during the current study are available from the corresponding author on reasonable request.
