# Supplementary material for: 44-year journey (1980–2024): scientometric insights into Sigesbeckiae herba and update on its medicinal properties and phytochemicals profile
Source: Chin Med. 2026 Mar 4;21:77. doi: 10.1186/s13020-025-01308-6 (PMC12958691; doi:10.1186/s13020-025-01308-6)
Supplement: Supplementary file 1 [file 13020_2025_1308_MOESM1_ESM.docx]

**Table 1**: Bioactivities shown by extracts/Compounds of various species of Sigesbeckiae Herba in different experimental models

| **Species/ Extract** | **Experimental Model** | **Dose/ IC_50_** | **Effects/ Mechanism** | **Citation** |
| --- | --- | --- | --- | --- |
| **Anti-inflammatory** | | | | |
| SH  Ethanolic extract | Adjuvant  arthritis model of rat | 4 mg/kg | AF*SH* could reduce the inflammatory pathologic response of ankle joint, it functions well as analgesics, with the analgesic rate being 65%, AF*SH* could also improve T-lymphocyte proliferation, improve IL-2 activity and inhibit IL-1 activity, as compared with the control group, the difference was significant (P < 0.01) | (Qian et al., 2000) |
| SP  Ent-16 αH, 17-hydroxy-kauran-19-oic-acid | (In vivo)  Carrageenan-induced oedema in rats | 20mg/ kg  30mg/kg | Inhibited the NO, COX-2 and THF-α production through.  down-regulating the NF-κB blinding activity | (Park et al., 2007) |
| SH  Aqueous extract | (In vivo)  Odium urate-induced acute gouty arthritis in mouse | 2.1 mg/kg  4.2 mg/kg  8.4 mg/kg | Inhibited the release of IL-1β and TNF-α decreased the expression of VCAM-1 | (G. C. Sun et al., 2007) |
| SO  MeOH extract | (In vivo) Xylene-induced inflammation in mice, Acetic acid caused writhing in mice | 5 mL/day | Reduced toe inflammation: reduced writhing frequency prolonged the time of licking hind feet. | (Luo et al., 2008) |
| SH  Kirenol | (In vivo) Collagen-induced arthritis in mice | 1mg/Kg  2mg/Kg  4mg/Kg | Upregulated the Annexin-1 to inhibit the NF-κB activity in a dose-dependent manner | (Z.-M. Wang et al., 2011) |
| SH  Aqueous &  ethanolic extract | (In vivo) Acute gouty arthritis in mice | 4.8 g/kg, 9.6 g/kg,  16.384 g/kg,  20.48 g/kg,  25.6 g/kg,  28 g/kg,  36 g/kg | Ethanolic extract was better than water extract in alleviating joint swelling (P< 0.05). | (Jiang et al., 2013) |
| SG  Methanolic extract | (In vivo) Ovalbumin-induced asthma murine in rats | 30mg/ kg | Decreased the production of IL-3 and COX-2 | (Teng, 2015) |
| SP  Aqueous extract | (In vivo) Iodoacetamide- induced colitis in rats | 12.5 mg/kg  25 mg/kg  50 mg/kg | Dose dependently decreased the expression of PPAR-γ | (Hong et al., 2014)] |
| SH  Aqueous extract | (In vivo) odium urate induced acute gouty arthritis in mice | 20 mg/g | Reduced the expression of IL-1β and IL-8 | (Qian et al., 2000) |
| SH  Aqueous extract | (In vivo) Sodium urate-induced gouty arthritis in mice | 1 g/kg,  2 g/kg,  4 g/kg | Different dosages of aqueous extract decreased the  expression of MyD88 and TRAF6 | (Li et al., 2019) |
| SH  Aqueous extract | (In vivo) Lupus nephritis in MRL/1pr mice | 500 mg/Kg | Reduced the IL-6 and TNF-α levels in the serum.  alleviated the inflammatory damage to the Kidney  pathological tissues | (Li et al., 2019) |
| SO  Aqueous extract | (In vivo) Knee osteoarthritis rat model | 2g/ kg | Alleviated cartilage injury by decreasing the level of  nicely-FOXO1 in cartilage tissue and increasing the  expression of sirt1 protein | (Tang et al., 2020) |
| SG  Glabriside C | In vitro | 40 μmol/L | anti-inflammatory properties in LPS-stimulated BV2 microglia, among these compounds, glabreside C (3) *SH*owed the most potent anti-inflammatory activity, dose-dependently promoted the production of HO-1, and inhibited the expression of iNOS and COX2 in LPS-activated BV2 cells. Mechanistically, glabreside C (3) could exert its anti-inflammatory effect by inhibiting the AKT/MAPKs signalling pathway, which presents a new chemical tool to probe the development of anti-inflammatory therapy | (Gao et al., 2021) |
| SO  Kirenol | In vitro MC3T3-E1 cells | 10 μM,  20 μM  40 μM | Dose-dependently increased the expression of  osteoblast differentiation markers (ALP, ColA1,  osteopontin), and stimulated the expression of BMP and  Wnt/β-catenin signaling pathways | (M.-B. Kim et al., 2014) |
| SH (SO, SP, SG) | invitro | 5-320ug/ mL | anti-inflammatory properties for the three main plant origins of *SH*. Although SG *SH*owed lower toxicity and less anti-inflammatory effects compared with SP and SO in LPS-induced RAW264.7 cells, comparable inhibitory effects on NF-κB and MAPKs pathways and the reduction of LPS-induced iNOS and COX-2 were observed in the anti-inflammatory process for all Sigesbeckia plants. | (Linghu et al., 2020) |
| Leocarpinolide B (From SG) | *In vivo*/*in vitro* | 2.5, 5.0, and 10.0 mg/kg | LB directly interacted with NF-κB p65 and reduced the DNA binding activity of NF-κB in synovial cells. In conclusion, LB significantly attenuated the collagen type II-induced arthritis, which was at least involved in the inhibition of DNA binding activity of NF-κB through a direct binding to NF-κB p65, LB could be a valuable lead compound for developing anti-RA drugs. | (Linghu et al., 2023) |
| **Antitumor/caner** | | | | |
| SG  Aqueous extract | (In vitro) MCF-7 cells, MDA- MB-231 cells |  | Induced apoptosis in MCF-7 cells through the endogenous signalling pathway; induced apoptosis in MCF-7 cells through the exogenous signalling pathway. | (Jun et al., 2006) |
| SO  Ethyl acetate & n-BuOH extract | (In vitro) HeLa cells | IC_50_= 200 μg/mL | Inhibited the proliferation of HeLa cells |  |
| SP  Ethanol extract | (In vitro) A549 cells | IC_50_= 58 μg/mL | Inhibited the proliferation of A549 cells via the  activation of p70s6k independent signalling pathway. | (Jia et al., 2012) |
| SO Ethanolic extract | In vitro (RL95-2 cells | IC_50_= 20 μg/mL | Inhibited TGFβ1-induced cell wound healing, cell  migration and cell invasion in RL95-2 cells. | (Chang et al., 2016) |
| SG Germacranolide | (In vitro) PANC-1 cells, AsPC-1 cells | IC_50_= 6.9 μM, 5.1 μ | Suppressed Gli-mediated transcriptional activity to  reduce cancer cell proliferation and downregulate  expression of the Gli-target genes, Gli1 and cyclin D1 in PANC-1 cells and  AsPC-1 cells | (H. J. Lee et al., 2016) |
| SP  Essential oil | (In vitro) Hep-G2 | IC_50_= 42 μg/mL | Increased the expression of Bax, caspase-3, and caspase-9.  decreased the expression of bcl-2 | (Lv et al., 2017) |
| **Antiallergic activity** | | | | |
| SG  Aqueous extract | (In vivo) LPS-stimulated murine whole spleen cells | 50 mg/kg,  100 mg/kg, 500  mg/kg, 1000 mg/kg | *SH*owed the inhibitory effect on IgE production from B  cells in a dose-dependent manner. | (Hwang et al., 2001) |
| SO  Ethanol extract | (In vivo) Ovalbumin-immunized mice | 0.25 mg,  0.5 mg,  1.0 mg | Suppressed the proliferation of splenocytes and  decreased the level of IgG, IgG1 and IgG2b in the serum | (H. Sun & Wang, 2006) |
| SH  Aqueous extract | (In vivo) Dinitrochlorobenzene-induced the  delayed-type hypersensitivity model  in rats | 3.75 g/kg,  7.50 g/kg,  15.0 g/kg | *SH*owed dose-dependently antiallergic activities regulated the immune functions by reducing the  antibody production inhibited the T-cell-mediated  delayed hypersensitivity reduced the phagocytic  ability and phagocytic index | (Shao et al., 2012) |
| SH  Kirenol | (In vivo) Experimental autoimmune  encephalomyelitis in rats | 2 mg/kg | Induced apoptosis in T cells via down-regulating interferon-gamma (IFN-γ) and IL-17A expression in  serum inhibited the differentiation of TH1 and TH17  cells increased caspase activity | (Xiao et al., 2015) |
| **Antibacterial activity** | | | | |
| SG  Methanolic extract | *S. aureus*  *B. subtilis* | MIC = 3.12, 6.25,  12.50 and 25.00  μg/mL | *SH*owed activity against S. aureus, B. Subtilis, S.  schleiferi, E. faecalis with the MIC values of 3.12, 6.25,  12.50 and 25.00 μg/mL, respectively | (Y.-S. Kim et al., 2012) |
| SH  Kirenol | *S. schleiferi*  *B. subtilis*  *S. aureus*  *S. epidermidis*  *S. oralis*  *A. baumannii*  *E. coli*  *(In vitro)*  *E. faecalis*  *P. aeruginosa* | MIC = 39.0, 78.0,  156.0, 39.0,  625.0, 312.5,  625.0 μg/mL | Kirenol *SH*owed the most excellent antibacterial activity  against B. subtilis and S. oralis with a MIC of 39.0 μg/mL.  The MIC of kirenol ranged from 39.0 to  625.0 μg/mL | (J.-P. Wang et al., 2012) |
| SH  Ent-17αH,18-  dihydroxy-kauran-19- oic acid | (In vitro) *C. Albicans* | MIC- 0.5 μg/mL  MBC= 1.0 μg/mL | Exhibited significant antibacterial activity against *C. albicans* | (Yang et al., 2016) |
| **Antioxidant activity** | | | | |
| SH  Aqueous extract | (In vivo) Doxorubicin-induced hepatic and renal injury in rats | 170 mg/kg  340 mg/kg | Improved the SOD and CAT activity, free radical  scavenging ability, and reduced the content of  peroxidation | (Yu et al., 2014) |
| SH  Aqueous extract | (In vitro) Synovial cells from rats | 1 g/kg,  2 g/kg,  4 g/kg | Dose-dependently reduced the ROS and MDA content in  synovial cells increased the Nrf2 mRNA expression. | (G. C. Sun et al., 2007) |
| SP | Invitro (HACAT cells) | 200 μg mL^−1^ | *SH*E protects cells from PM_10_ toxicity by increasing the cellular antioxidant capacity and that chlorogenic acid may be an active phytochemical of *SH*E. | (J.-P. Wang et al., 2012) |
| **Antithrombotic activity** | | | | |
| SH  70 % Ethanolic extract | (In vivo) Blood stasis model in rats | 55 g/kg,  110 g/kg | Reduced plasma fibrinogen content and inhibited the  endothelin secretion | (K. Lee et al., 2013) |
| SH  Ent-16β,17-  dihydroxykauran-19-  oic acid | (In vivo) arterio-venous *SH*unt model | 200 μg/mL,  400 μg/mL,  600 μg/mL,  800 μg/mL, 1000 μg/mL | Reduced AVB thrombus weight and increased cAMP  level; inhibited various agonists, stimulated platelet  aggregation and caused an increase in cAMP levels in  platelets activated by ADP | (Meng et al., 2009) |
| SH  Aqueous extract | Blood from beagle dogs | 250 mg/kg,  500 mg/kg, 1000 mg/kg | High dose (1000 mg/kg) of *SH* aqueous extract reduced  the platelet aggregation rate, hematocrit value, whole  blood and plasma viscosity accelerated the erythrocyte  sedimentation rate, and prolonged the thrombin time  and prothrombin time. | (Y. Wang et al., 2012) |
| SH | Molecular docking technique |  | Down-regulated serum ALB, F2, F10 and ITGA2B  expression to prevent platelet aggregation | [50] |
| SP  n- butanol fraction | (In vivo) MTT assay, TBA and ROS assay, GSSG, western blotting and qRT-PCR | 50 μg mL^−1^ | can increase the cellular antioxidant capacity through the induction of defence genes, such as GCL-c, GCL-m, and G6PDH, and mitigate oxidative stress and enhance cell viability under PM_10_-exposed conditions | (Ha & Boo, 2021) |
| **Cerebral ischemia injury protective activity** | | | | |
| SH  Aqueous extract | (In vivo) Acute incomplete cerebral ischemia in  rats | 200 mg/kg, 400  mg/kg,  800 mg/kg | Reduced brain water content, brain index, and  cerebral vascular permeability in a dose-dependent  manner. | (Z.-M. Wang et al., 2011) |
| SO  Ethanolic extract | (In vivo) Acute myocardial ischemia model in  rats | 7g/kg | The cerebral blood flow in EB-treated group decreased after the ischemia-reperfusion injury. In the *SH*-treated group, the antioxidase activity increased, and the content of malondialodehyde decreased | (Xiao et al., 2015) |
| SH  ,3′ -dihydroxy-3,7,4′ -  trimethoxyflavone | (In vitro) Mouse hippocampal HT22 cell | One μM,  5 μM,  10 μM,  20 μM,  40 μM | Dose-dependently protected the nerve cells through the  regulation of P13k-Akt and brain-derived neurotrophic  factor signalling pathways, such as upregulating the  expression of CDK5, MAPK10 and MDM2 | (Han et al., 2012) |
| SH  Aqueous extract | Invitro (MCAO rats) |  | Herba siegesbeckiae extract treatment significantly decreased mNSS score and infarct volume in ischemic cerebral infarction in the model group. It increased Nissl-positive cells and NeuN expression, reduced Tunel-positive cells, inhibited Bax expression, increased Bcl-2 and NeuN in ischemic brain tissue, and inhibited IL-1β, TNF-α, and IL-6 mRNA expression. | (Xiao et al., 2015) |
| **Myocardial injury protective activity** | | | | |
| SH  Aqueous extract | (In vivo) Rat thoracic aorta ring methods | 2 mg/mL,  4 mg/mL,  6 mg/mL,  8 mg/mL,  10 mg/mL,  12 mg/mL | Inhibited the contraction of blood vessels induced by  phenylephrine, related to endothelial NO-dependent  vasodilation effect of the aqueous extract of *SH* | (Yang & LU, 2007) |
| SH  Ethanolic extract | (In vivo) Pressure overload-induced  myocardial remodelling | 4.8 g/kg,  9.6 g/k | A high dose of *SH* ethanol extract reduced diastolic blood  pressure and systolic pressure of model mice and  improved heart weight index and hemodynamic | (Wei-wei et al., 2011) |
| SH  Ethanolic extract | (In vivo) Doxorubicin-induced chronic  myocardial injury in rats | 340 mg/kg | Protected the myocardial injury caused by doxorubicin | (Yu et al., 2013) |
| SH  Kirenol | (In vivo) Diabetic heart in rats’ model | 20 μmol/L,  40 μmol/L | Attenuated activation of MAPK subfamily, NF-κB,  Smad2/3, SP1 and AP-1, and decreased phosphorylation of Iκ -Bα | (Wu et al., 2019) |
| **Antidiabetic** | | | | |
| SG  Ent-16,17-  isobutyryloxy-kauran-19-oic acid | (In vitro) PTP1B | IC50 = 8.7 ± 0.9  μg/mL | Lower blood glucose, and was non-competitive  inhibitory of PTP1B | (Z.Y. Li, 2007) |
| SO  Aqueous extract,  ethanol extract | (In-vivo) Streptozotocin-induced diabetes in rats | 500 mg/kg,  500 mg/kg. | Two extracts improved sugar tolerance and insulin-  resistance. The effect of aqueous extract was better than  that of ethanol extract, | (D.Y. Li, 2007) |
| **Uricosuric agents** | | | | |
| SH  Aqueous extract | (In vivo) adenine/oxazine acid-induced  hyperuricemia mice model | 2.1 g/kg,  4.2 g/kg,  8.4 g/kg | Middle and high doses of the extract were better than  allopurinol in reducing the uric acid, urea nitrogen, and  creatinine levels in serum through activating urate  oxidase and suppressing purine oxidase activity | (D.Y. Li, 2007) |
| **Fibroblast stimulator** | | | | |
| SO  Methanolic extract | (In vitro) Mouse dermal fibroblasts | 1.0 μg/mL,  50 μg/mL,  100 μg/mL,  200 μg/mL | 50–200 μg/mL methanolic extract of S. orientalis  *SH*owed significant proliferative action of fibroblasts,  accelerated the repair of skin damage. | (Q. Luo et al., 2008) |
| **Skin whitening (Cosmetic)** | | | | |
| SG | In vivo/ invitro | 0.1–20 μg/mL | SGMFAb positively influences skin whitening activities by inhibiting melanogenesis and melanosome-transport-related events in B16BL6 cells and suggest that SGMFAb is a promising material for developing functional skin whitening agents. | (Hughes et al., 2019) |

* SH: Sigesbeckiae Herba; SO: *S. orientalis* L.; SP: S. *pubescens* Makino; SG: *S. glabrescens* Makino.

**References**

Chang, C.-C., Ling, X.-H., Hsu, H.-F., Wu, J.-M., Wang, C.-P., Yang, J.-F., Fang, L.-W., & Houng, J.-Y. (2016). *Siegesbeckia orientalis* extract inhibits TGFβ1-induced migration and invasion of endometrial cancer cells. *Molecules*, *21*(8), 1021.

D.Y. Li. (2007). , Effect of prescription Herba Siegesbeckiae capsule on rats hyperuricemia and uratic renal injury . *Heilong Jiang Univ. Chin. Med*.

Gao, X., Shen, X., Zheng, Y., Yang, L., Zhang, X., Hu, G., Jia, J., & Wang, A. (2021). Sesquiterpene lactones from *Sigesbeckia glabrescens* possessing potent anti-inflammatory activity by directly binding to IKKα/β. *Journal of Natural Products*, *84*(11), 2808–2821.

Ha, J. W., & Boo, Y. C. (2021). Siegesbeckiae herba extract and chlorogenic acid ameliorate the death of HaCaT keratinocytes exposed to airborne particulate matter by mitigating oxidative stress. *Antioxidants*, *10*(11), 1762.

Han, L., Zhou, X. H., & Wang, W. W. (2012). Observation on effect of Herba Siegesbeckiae on antioxidase during cerebral ischemia-reperfusion injury in mice. *Chin. Arch. Tradit. Chin. Med*, *30*(10), 2287–2289.

Hong, Y.-H., Weng, L.-W., Chang, C.-C., Hsu, H.-F., Wang, C.-P., Wang, S.-W., & Houng, J.-Y. (2014). Anti‐Inflammatory Effects of *Siegesbeckia orientalis* Ethanol Extract in In Vitro and In Vivo Models. *BioMed Research International*, *2014*(1), 329712.

Hughes, K., Ho, R., Butaud, J.-F., Filaire, E., Ranouille, E., Berthon, J.-Y., & Raharivelomanana, P. (2019). A selection of eleven plants used as traditional Polynesian cosmetics and their development potential as anti-aging ingredients, hair growth promoters and whitening products. *Journal of Ethnopharmacology*, *245*, 112159. https://doi.org/10.1016/j.jep.2019.112159

Hwang, W.-J., Park, E.-J., Jang, C.-H., Han, S.-W., Oh, G.-J., Kim, N.-S., & Kim, H.-M. (2001). Inhibitory effect of immunoglobulin E production by jin-deuk-chal (*Siegesbeckia orientalis*). *Immunopharmacology and Immunotoxicology*, *23*(4), 555–563.

Jia, L. I. U., Rong, C., Yu, N. I. E., Lin, F., & Hai-Dao, L. I. (2012). A new carbamate with cytotoxic activity from the aerial parts of *Siegesbeckia pubecens*. *Chinese Journal of Natural Medicines*, *10*(1), 13–15.

Jiang, F. P., Fu, X. C., & Bai, H. B. (2013). Mice acute toxicity of Siegesbeckia and its effect on mouse acute gouty arthritis. . *Chin. JMAP*, *30*(12), 1289–1291.

Jun, S. Y., Choi, Y. H., & Shin, H. M. (2006). *Siegesbeckia glabrescens* induces apoptosis with different pathways in human MCF-7 and MDA-MB-231 breast carcinoma cells. *Oncology Reports*, *15*(6), 1461–1467.

Kim, M.-B., Song, Y., & Hwang, J.-K. (2014). Kirenol stimulates osteoblast differentiation through activation of the BMP and Wnt/β-catenin signaling pathways in MC3T3-E1 cells. *Fitoterapia*, *98*, 59–65.

Kim, Y.-S., Kim, H., Jung, E., Kim, J.-H., Hwang, W., Kang, E.-J., Lee, S., Ha, B.-J., Lee, J., & Park, D. (2012). A novel antibacterial compound from *Siegesbeckia glabrescens*. *Molecules*, *17*(11), 12469–12477.

Lee, H. J., Wu, Q., Li, H., Bae, G.-U., Kim, A. K., & Ryu, J.-H. (2016). A sesquiterpene lactone from *Siegesbeckia glabrescens* suppresses Hedgehog/Gli-mediated transcription in pancreatic cancer cells. *Oncology Letters*, *12*(4), 2912–2917.

Lee, K., Jung, J., Yang, G., Ham, I., Bu, Y., Kim, H., & Choi, H. (2013). Endothelium‐independent vasorelaxation effects of *Sigesbeckia glabrescens* (makino) makino on isolated rat thoracic aorta. *Phytotherapy Research*, *27*(9), 1308–1312.

Li, Q., W, Y. L. H., & Yu, Q. H. (2019). Therapeutic effect of Siegesbeckiae Herba on lupus nephritis in MRL/lpr mice. *Chin. Med. J. Re.s Prac*, *33*(4), 12–16.

Linghu, K.-G., Zhao, G. D., Xiong, W., Sang, W., Xiong, S. H., Tse, A. K. W., Hu, Y., Bian, Z., Wang, Y., & Yu, H. (2020). Comprehensive comparison on the anti-inflammatory effects of three species of Sigesbeckia plants based on NF-κB and MAPKs signal pathways in vitro. *Journal of Ethnopharmacology*, *250*, 112530.

Linghu, K.-G., Zhao, G.-D., Zhang, D.-Y., Xiong, S.-H., Wu, G.-P., Shen, L.-Y., Cui, W.-Q., Zhang, T., Hu, Y.-J., & Guo, B. (2023). Leocarpinolide B attenuates collagen type II-induced arthritis by inhibiting DNA binding activity of NF-κB. *Molecules*, *28*(10), 4241.

Luo, Q., Wang, J. P., Ruan, J. L., Chen, Y. A., & Li, Y. Y. (2008). Research on anti-inflammatory and analgesic effect of local application of *Siegesbeckia orientalis* L. *J Hubei, Coll. Tradit. Chin. Med*, *10*(3), 9–11.

Lv, D., Guo, K., Xu, C., Huang, M., Zheng, S., Ma, X., Pan, L., Wang, Q., & Yang, X. (2017). Essential oil from *Siegesbeckia pubescens* induces apoptosis through the mitochondrial pathway in human HepG2 cells. *Journal of Huazhong University of Science and Technology [Medical Sciences]*, *37*, 87–92.

Meng, Q. C., Jin, R. M., Wang, D., & Chou, G. X. (2009). ffects of antithrombotic components of Herba Siegesbeckiae on coagulation system,. *Shanxi J. Tradit. Chin. Med*, *30*(2), 236–238.

Park, H.-J., Kim, I.-T., Won, J.-H., Jeong, S.-H., Park, E.-Y., Nam, J.-H., Choi, J., & Lee, K.-T. (2007). Anti-inflammatory activities of ent-16αH, 17-hydroxy-kauran-19-oic acid isolated from the roots of *Siegesbeckia pubescens* are due to the inhibition of iNOS and COX-2 expression in RAW 264.7 macrophages via NF-κB inactivation. *European Journal of Pharmacology*, *558*(1–3), 185–193.

Q. Luo, J.P. Wang, J.L. Ruan, Y.A. Chen, & Y.Y. Li. (2008). , Promotion of the cutaneous wound healing  by *Siegesbeckiae orientalis* L,. *Herald Med,* , *27*(10), 1161–1163.

Qian, R., Zhang, C., & Fu, H. (2000). Study on therapeutic mechanism of anti-rheumatism action of herba siegesbeckiae. *Zhongguo Zhong Xi Yi Jie He Za Zhi Zhongguo Zhongxiyi Jiehe Zazhi= Chinese Journal of Integrated Traditional and Western Medicine*, *20*(3), 192–195.

Shao, N. Q., Zhao, M., Wang, H. Q., & Zhu, X. X. (2012). Experimental research of effect of herba siegesbeckiae on immunological function of mice. *Chin Arch Tradit Chin Med*, *8*, 66.

Sun, G. C., Yu, X. F., & Li, D. Y. (2007). Compound Xiqiancao (siegesbeckiae) capsule on content of IL-1β, IL-8 in rats’ inflammatory articulus caused by sodium urate. *World Journal of Integrated Traditional and Western Medicine*, *2*(6), 329–331.

Sun, H., & Wang, H. (2006). Immunosuppressive activity of the ethanol extract of *Siegesbeckia orientalis* on the immune responses to ovalbumin in mice. *Chemistry & Biodiversity*, *3*(7), 754–761.

Tang, X.-D., Zhao, Q., Lan, X.-F., Ge, N.-N., Tang, Z.-H., & Fan, C.-H. (2020). Effect of *Siegesbeckia orientalis* on cartilage damage in knee osteoarthritis rats by regulating sirt1/FOXO1 pathway. *Chinese Journal of Immunology*, *36*, 439–444.

Teng, T. L. (2015). Study on effect and mechanism of the aqueous extract from *Siegesbeckiae pubescens* on ulcerative colitis in rats. *Zhejiang Acad. Med. Sci*.

Wang, J.-P., Zhou, Y.-M., & Zhang, Y.-H. (2012). Kirenol production in hairy root culture of *Siegesbeckea orientalis* and its antimicrobial activity. *Pharmacognosy Magazine*, *8*(30), 149.

Wang, Y., Li, B. Q., Zhou, L., Zhao, H., & Gao, J. Y. (2012). Effect of Herba Siegesbeckiae capsule on coagulation function and hemorheology in beagle dogs. *Asia-Pacific, Tradit. Med*, *8*(3), 6–7.

Wang, Z.-M., Zhu, S.-G., Wu, Z.-W., Lu, Y., Fu, H.-Z., & Qian, R.-Q. (2011). Kirenol upregulates nuclear annexin-1 which interacts with NF-κB to attenuate synovial inflammation of collagen-induced arthritis in rats. *Journal of Ethnopharmacology*, *137*(1), 774–782.

Wei-wei, W., Lei, H., Xiao-hui, Z., Tian-jiao, S., & Shi-bin, Z. (2011). Influence of Siegesbeckia on Myocardial Remodeling in Rats with Pressure Overload. *JOURNAL OF LIAONING UNIVERSITY OF TCM*, *13*(7), 102–105.

Wu, B., Huang, X., Li, L., Fan, X., Li, P., Huang, C., Xiao, J., Gui, R., & Wang, S. (2019). Attenuation of diabetic cardiomyopathy by relying on kirenol to suppress inflammation in a diabetic rat model. *Journal of Cellular and Molecular Medicine*, *23*(11), 7651–7663.

Xiao, J., Yang, R., Yang, L., Fan, X., Liu, W., & Deng, W. (2015). Kirenol attenuates experimental autoimmune encephalomyelitis by inhibiting differentiation of Th1 and th17 cells and inducing apoptosis of effector T cells. *Scientific Reports*, *5*(1), 9022.

Yang, Y., Chen, H., Lei, J., & Yu, J. (2016). Biological activity of extracts and active compounds isolated from *Siegesbeckia orientalis* L. *Industrial Crops and Products*, *94*, 288–293.

Yang, Y., & LU, J. (2007). Experimental Study on Endothelial NO-dependent Vasodilation Effect of the Extracts of Herba Siegesbeckiae. *China Pharmacy*.

Yu, J., Wang, J., Su, S. W., Xie, K. R., & Zhang, Y. (2013). Protective role of Herba Siegesbeckiae extracts against doxorubicin-induced acute cardiac injury in rats, . *Herald Med*, *13*(7), 843–847.

Yu, J., Wang, J. X., Su, S. W., Xie, K. R., Zhang, Y., Yang, J. Z., & Su, Y. (2014). Preventive role of extracts from Herba Siegesbeckiae against acute hepatic and renal injury induced by doxorubicin in rats. *Herald Med*, *33*(4), 422–426.

Z.Y. Li. (2007). Study on Hypoglycemic Effect of Herba Siegesbeckiae and Cortex Mori Radicis. *Yanbian Univ* .
