# Supplementary material for: 44-year journey (1980–2024): scientometric insights into Sigesbeckiae herba and update on its medicinal properties and phytochemicals profile
Source: Chin Med. 2026 Mar 4;21:77. doi: 10.1186/s13020-025-01308-6 (PMC12958691; doi:10.1186/s13020-025-01308-6)
Supplement: Supplementary file 2 [file 13020_2025_1308_MOESM2_ESM.docx]

**Supplementary Table 2**: All the compounds identified from three species of Sigesbeckia plants (SO: *Sigesbeckia orientalis* L.; SP: *S. pubescens* Makino; and SG: *S. glabrescens* Makino).

| **Species** | **Compound Name** | **Class of Compound** |
| --- | --- | --- |
| SP | 3,3',4'-Trimethoxy quercetin | flavonoid |
| SP | 3,3'-​Dimethoxy quercetin | flavonoid |
| SP | Quercetin | flavonoid |
| SP | Kaempferol-​3-​β-​D-​glucoside | flavonoid |
| SP | Isorhamnetin | flavonoid |
| SP | Quercetin-​3-​β-​D-​glucoside | flavonoid |
| SP | Kaempferol | flavonoid |
| SP | Quercetin-​3-​O-​α-​L-​rhamnoside | flavonoid |
| SP | Myricetin | flavonoid |
| SP | 3',4'-Dimethoxy quercetin | flavonoid |
| SG | 3,​7,​4'-​O-​Trimethylquercetin | flavonoid |
| SG | 3,​4'-​O-​Dimethylquercetin | flavonoid |
| SO, SP | Rutin | flavonoid |
| SP, SG | 3-​Methylquercetin | flavonoid |
| SO, SP, SG | 3',7-Dimethylguercetin | flavonoid |
| SP | 7,3',4'-​Trimethoxy luteolin | flavonoid |
| SP | Luteolin | flavonoid |
| SP | Apigenin | flavonoid |
| SP | Vitexin | flavonoid |
| SP | Luteoloside | flavonoid |
| SP | Baicalin | flavonoid |
| SP | Vicenin-​2 | flavonoid |
| SP | 2''-O-Rhamnosyl vitexin | flavonoid |
| SP | Chrysin-8-C-β-D-glucoside | flavonoid |
| SP | Hesperidoside | flavonoid |
| SP | Liquiritin | flavonoid |
| SP | 5',7'-Dihydroxy-4'-glucosyloxyisoflavone | flavonoid |
| SP | Isoliquiritin | flavonoid |
| SP | 3',5', β-Trihydroxy-3,4,4',α-tetramethoxy-chalcone | flavonoid |
| SO, SG | Siegesesteric acid I | kaurenoids |
| SO | Siegesesteric acid II | kaurenoids |
| SO, SP, SG | Annosquamosin A | kaurenoids |
| SP | Ent-17,18-dihydroxy-16βH-kauran-19-oic acid | kaurenoids |
| SP | Ent-16α,17,18-trihydroxykauran-19-oic acid | kaurenoids |
| SP | Ent-16αH-kauran-17,19-dioic acid | kaurenoids |
| SP, SG | Siegeskaurolic acid | kaurenoids |
| SO, SP | Ent-​16β,​17-​dihydroxykauran-​19-​oic acid-​16β,​17-​acetonide | kaurenoids |
| SP | 16α,17-Acetonide of ent-19-methyl-16α,17-dihydroxykauran-19-oic acid | kaurenoids |
| SP | Siegesbeckiol | kaurenoids |
| SP | Ent-kauran-16β,17,18-triol | kaurenoids |
| SG | Kaurane-16,17,18-triol | kaurenoids |
| SP | Kauralexin A2 | kaurenoids |
| SP | (-)-17-Hydroxy-16α-kauran-19-oic acid | kaurenoids |
| SP | Ent-17-hydroxy-16βH-kauran-19-oic acid | kaurenoids |
| SP | Ent-18-acetoxy-17-isobutyryloxy-16βH-kauran-19-oic acid | kaurenoids |
| SP | Ent-18-acetoxy-16α-hydroxy-17-isobutyryloxykauran-19-oic acid | kaurenoids |
| SP | Ent-17-isobutyryloxy-18-hydroxykauran-19-oic acid | kaurenoids |
| SP, SG | Ent-​16βH,17-​isobutyryloxy-​kauran-​19-​oic acid | kaurenoids |
| SP | Ent-18-acetoxy-16α,17-dihydroxykauran-19-oic acid | kaurenoids |
| SP | Ent-18-acetoxy-17-hydroxy-16βH-kauran-19-oic acid | kaurenoids |
| SP | (4α)​-18-​(Acetyloxy)​-​17-​hydroxy-norkaurane-​4,​16-​dicarboxylic acid | kaurenoids |
| SP, SG | Grandifloric acid | kaurenoids |
| SP | Ent-19-methyl-17-hydroxy-16αH-kauran-19-oic acid | kaurenoids |
| SP | Ent-16α,17-dihydroxy-kauran-19-oic acid methyl ester | kaurenoids |
| SP | Ent-​16α,17-​dihydroxy-​kauran-​19-​oic acid | kaurenoids |
| SP | Ent-16β,17,18-trihydroxykauran-19-oate methyl | kaurenoids |
| SP | Ent-17-hydroxy-16α-kauran-19-oic acid methyl ester | kaurenoids |
| SP | 2-O-[β-D-Apiofuranosyl-(1→3)-2-O-isovaleryl-β-D-glucopyranosyl]-4-epi-atractyligenin | kaurenoids |
| SP | 2-O-[β-D-Apiofuranosyl-(1→3)-2-O-isovaleryl-β-D-glucopyranosyl]atractyligenin | kaurenoids |
| SP | 2-O-[β-D-Apiofuranosyl-(1→3)-2-O-(3-methylpentanoyl)-β-D-glucopyranosyl]-4-epi-atractyligenin | kaurenoids |
| SP | 2-O-(2-O-Isovaleryl-β-D-glucopyranosyl)-4-epi-atractyligenin | kaurenoids |
| SP, SG | Seigesbeckioside | kaurenoids |
| SG | 16β,​17-​Dihydroxy- kaurane | kaurenoids |
| SG | (4β)​-19-Hydroxy-​kaur-​16-​en-​18-​oic acid | kaurenoids |
| SP | Siegeside E | kaurenoids |
| SO | 18-*O*-acetyl-kauran-16-ent-19-oic acid | kaurenoids |
| SO | Sigesbeckin B | kaurenoids |
| SO | Sigesbeckin C | kaurenoids |
| SO | Ent-​2α,​15,​16,​19-​tetrahydroxypimar-​8(14)​-​ene | pimarane diterpenoid |
| SO | 2β,15,16-Trihydroxy-ent-pimar-8(14)-ene | pimarane diterpenoid |
| SO | 15,16-Dthydroxy-2-oxo-ent-pimar-8(14)-ene | pimarane diterpenoid |
| SO | Ent-​2-​oxo-​15,​16-​dihydroxypimar-​8(14)​-​en-​16-​O-​β-​glucopyranoside | pimarane diterpenoid glucoside |
| SP | Siegeside B aglycon | pimarane diterpenoid |
| SP | Siegeside A | pimarane diterpenoid glucoside |
| SO | 15,16,18-Trihydroxy-2-oxo-ent-pimar-8(14)-ene | pimarane diterpenoid |
| SO | Orientalin A | pimarane diterpenoid |
| SO, SP, SG | Orientalin B | pimarane diterpenoid |
| SO, SP, SG | Kirenol | pimarane diterpenoid |
| SO | Ent-15-oxo-2β,16,19-trihydroxypimar-8(14)-ene | pimarane diterpenoid |
| SO, SP | Ent-2-oxo-15,16,19-trihydroxypimar-8(14)-ene | pimarane diterpenoid |
| SP | Ent-2α,7α,15,16,19-pentahydroxypimar-8(14)-ene | pimarane diterpenoid |
| SP | Ent-2α,15,16,18-tetrahydroxypimar-8(14)-ene | pimarane diterpenoid |
| SO | 9β-​Hydroxydarutigenol | pimarane diterpenoid |
| SO | 16-​O-​Acetyldarutigenol | pimarane diterpenoid |
| SO, SP | Ent-3α,7β,15,16-tetrahydroxypimar-8(14)-ene | pimarane diterpenoid |
| SO, SP, SG | Darutigenol | pimarane diterpenoid |
| SO, SP, SG | Darutoside | pimarane diterpenoid glucoside |
| SP | Siegeside B | pimarane diterpenoid glucoside |
| SO | Ent-2β,15,16,19-tetrahydroxy-pimar-8(14)-en-19-O-β-glucopyranoside | pimarane diterpenoid glucoside |
| SO, SP | Pubeside A | pimarane diterpenoid glucoside |
| SO, SP | Pubeside B | pimarane diterpenoid glucoside |
| SO, SP | Pubeside C | pimarane diterpenoid glucoside |
| SO, SP | Pubeside D | pimarane diterpenoid glucoside |
| SO | β-​D-​Glucopyranosyl-​ent-​2-​oxo-​15,​16-​dihydroxy-​pimar-​8(14)​-​en-​19-​oic-​late | pimarane diterpenoid glucoside |
| SO, SP | Hythiemoside A | pimarane diterpenoid glucoside |
| SO | Hythiemoside B | pimarane diterpenoid glucoside |
| SO | Ent-​2-​oxo-​3β,​15,​16-​trihydroxy-pimar-​8(14)​-​en-​3-​O-​β-​glucopyranoside | pimarane diterpenoid glucoside |
| SO | 15,​16-​Di-​O-​acetyldarutoside | pimarane diterpenoid glucoside |
| SO | Ent-​12α,​16-​epoxy-​2β,​15α,​19-​trihydroxypimar-​8(14)​-​ene | pimarane diterpenoid |
| SO | Ent-​14β,​16-​epoxy-​8-​pimarene-​3β,​15α-​diol | pimarane diterpenoid |
| SP | 14β,16-Epoxy-ent-3β,15α,19-trihydroxypimar-7-ene | pimarane diterpenoid |
| SP | 14β,16-Epoxy-ent-3α,15α,19-trihydroxypimar-7-ene | pimarane diterpenoid |
| SO | Ent-14β,16-epoxy-8-pimarene-2α,15α,19-triol | pimarane diterpenoid |
| SO, SP, SG | Isopropylidenekireno​l | pimarane diterpenoid |
| SP | Pubeside E | pimarane diterpenoid glucoside |
| SP | Ent-3α,15,16-trihydroxypimar-8(14)-en-15,16-acetonide | pimarane diterpenoid |
| SP | Ent-3α,15,16-trihydroxypimar-8(14)-en-3α-O-β-glucopyranoside-15,16-acetonide | pimarane diterpenoid glucoside |
| SP | (1R,​3S,​4aS,​4bR,​7S,​10aS)​-7-​(2,​2-​Dimethyl-​1,​3-​dioxolan-​4-​yl)​-​1,​2,​3,​4,​4a,​4b,​5,​6,​7,​9,​10,​10a-​dodecahydro-​3-​hydroxy-​1,​4a,​7-​trimethyl-1-​phenanthrenemethanol​ | pimarane diterpenoid |
| SP | 13α-(1,2-Dihydroxyethyl)-13-methyl-5β,9β,10α-podocarp-8(14)-ene-6β,16-diol | pimarane diterpenoid |
| SP | Ent-2β,15,16-trihydroxypimar-8(14)-en-19-oic acid | pimarane diterpenoid |
| SP | (1R,​3S,​4aS,​4bR,​7S,​10aS)​-7-​(1,​2-​Dihydroxyethyl)​-​1,​2,​3,​4,​4a,​4b,​5,​6,​7,​9,​10,​10a-​dodecahydro-​3-​hydroxy-​1,​4a,​7-​trimethyl-1-​phenanthrenecarboxyl​ic acid | pimarane diterpenoid |
| SP | Ent-3α,15,16,19-tetrahydroxypimar-8(14)-ene | pimarane diterpenoid |
| SP | 1-​[(2S,​4aR,​4bS,​7R,​8R,​8aS)​-​2,​3,​4,​4a,​4b,​5,​6,​7,​8,​8a,​9,​10-​Dodecahydro-​7-​hydroxy-​8-​(hydroxymethyl)​-​2,​4b,​8-​trimethyl-​2-​phenanthrenyl]​-1,​2-​ethanediol | pimarane diterpenoid |
| SP | Ent-2α,3β,15,16,19-pentahydroxypimar-8(14)-ene | pimarane diterpenoid |
| SP | 1-​[(2S,​4aR,​4bS,​6S,​8R,​8aS)​-​2,​3,​4,​4a,​4b,​5,​6,​7,​8,​8a,​9,​10-​Dodecahydro-​6-​hydroxy-​8-​(hydroxymethyl)​-​2,​4b,​8-​trimethyl-​2-​phenanthrenyl]​-1,​2-​ethanediol | pimarane diterpenoid |
| SP | (1R,​4aS,​4bR,​7S,​10aS)​-7-​(1,​2-​Dihydroxyethyl)​-​1,​4,​4a,​4b,​5,​6,​7,​9,​10,​10a-​decahydro-​1-​(hydroxymethyl)​-​1,​4a,​7-​trimethyl-​3(2H)​-​phenanthrenone | pimarane diterpenoid |
| SP | (2R,​4aS,​4bR,​7S,​10aS)​-​7-​[2-​(Acetyloxy)​-​1-​hydroxyethyl]​-​1,​2,​3,​4,​4a,​4b,​5,​6,​7,​9,​10,​10a-​dodecahydro-​1,​1,​4a,​7-​tetramethyl-​2-​phenanthrenyl-3-β-​D-​glucopyranoside | pimarane diterpenoid glucoside |
| SP | Ent-2α,7β,15,16,19-pentahydroxypimar-8(14)-ene | pimarane diterpenoid |
| SP | Ent-2α,15R,16,19- tetrahydroxypimar-8(14)-ene | pimarane diterpenoid |
| SP | Ent-2β,15,16,19-tetrahydroxypimar-8(14)-ene | pimarane diterpenoid |
| SP | Ent-3β,15,16-trihydroxypimar-6,8(14)-diene | pimarane diterpenoid |
| SP | Ent-2α,15,16, 19-tetrahydroxypimar-6,8(14)-diene | pimarane diterpenoid |
| SP | 1-​[(2S,​4aR,​4bS,​7R,​8aS)​-​2,​3,​4,​4a,​4b,​5,​6,​7,​8,​8a,​9,​10-​Dodecahydro-​7-​hydroxy-​2,​4b,​8,​8-​tetramethyl-​2-​phenanthrenyl]​-1,​2-​ethanediol | pimarane diterpenoid |
| SP | (2R,​4aS,​4bR,​7S,​10aS)​-​7-​(1,​2-​Dihydroxyethyl)​-​1,​2,​3,​4,​4a,​4b,​5,​6,​7,​9,​10,​10a-​dodecahydro-​1,​1,​4a,​7-​tetramethyl-​2-​phenanthrenyl-3-β-​D-​glucopyranoside | pimarane diterpenoid glucoside |
| SP | Ent-1β,3β,15,16-tetrahydroxypimar-8(14)-ene | pimarane diterpenoid |
| SP | 2-(1,2-Dihydroxyethyl)-2,3,4,4a,4b,5,6,7,8,8a,9,10-dodecahydro-2,4b,8,8-tetramethyl-9-phenanthrenyl-6-β-D-glucopyranoside | pimarane diterpenoid glucoside |
| SP | Ent-​16-​nor-​3-​oxo-​pimar-​8(14)​-​en-​15-​al | pimarane diterpenoid |
| SP | Ent-15,16-dihydroxy-18-norpimar-8(14)-en-3-one | pimarane diterpenoid |
| SP | Ent-15,16-dihydroxypimar-1,8(14)-dien-3-one | pimarane diterpenoid |
| SP | Ent-16-acetoxy-3α,15-dihydroxy-14α-hydroperoxypimar-7-en-3α-O-β-glucopyranoside | pimarane diterpenoid glucoside |
| SG | Neodarutoside | pimarane diterpenoid glucoside |
| SP | Siegeside C | pimarane diterpenoid glucoside |
| SP | Siegeside D | pimarane diterpenoid glucoside |
| SP | Strobols A | strobane diterpenoid |
| SP | Strobols B | strobane diterpenoid |
| SP | β-glucopyranosyl-18-acetoxy-16a, 17 dihydroxykauran-19-oate | *ent*-pimarane diterpenoid |
| SP | 15-O-malonylkirenol | *ent*-pimarane diterpenoid |
| SP | 16-O-malonylkirenol | *ent*-pimarane diterpenoid |
| SG | 15,16-di-*O*-malonylkirenol | *ent*-pimarane diterpenoid |
| SG | Glabreside A | *ent*-pimarane diterpenoid |
| SG | Glabreside B | *ent*-pimarane diterpenoid |
| SG | Glabreside C | *ent*-pimarane diterpenoid |
| SG | siegeside Q | *ent*-pimarane diterpenoid |
| SG | siegeside R | *ent*-pimarane diterpenoid |
| SG | siegeside S | *ent*-pimarane diterpenoid |
| SG | siegeside T | *ent*-pimarane diterpenoid |
| SG | siegeside U | *ent*-pimarane diterpenoid |
| SG | siegeside V | *ent*-pimarane diterpenoid |
| SG | Sigesbeckia K | *ent*-pimarane diterpenoid |
| SG | Sigesbekia L | *ent*-pimarane diterpenoid |
| SO | Leocarpinolide F | sesquiterpenoid |
| SO | 2-Methylbut-2-enoic acid (*3aS,4S,5S,6Z,10Z,11aR*)-5-(acetyloxy)-2,3,3a,4,5,8,9,11a-octahydro-6,10bis(hydroxymethyl)-3-methylene-2-oxo-cyclodeca[b]furan-4-yl ester | sesquiterpenoid |
| SO | [*1(10)​E,​4Z*]-​8β-​(Angeloyloxy)​-​9α-​ethoxy-​6α,​15-​dihydroxy-​14-​oxogermacra-​1(10)​,​4,​11(13)​-​trien-​12-​oic acid 12,​6-​lactone | sesquiterpenoid |
| SO | [*1(10)​E,​4Z*]​-​8β-​Angeloyloxy-​9α-​methoxy-​6α,​15-​dihydroxy-​14-​oxogermacra-​1(10)​,​4,​11(13)​-​trien-​12-​oic acid 12,​6-​lactone | sesquiterpenoid |
| SO | [*3aS-​(3aR*,​4R*,​5S*,​6E,​10E,​11aS*)*​]​-2-Propenoic acid​, 2-​methyl-​, 2,​3,​3a,​4,​5,​8,​9,​11a-​octahydro-​5-​hydroxy-​6,​10-​dimethyl-​3-​methylene-​2-​oxocyclodeca[b]​furan-​4-​yl ester | sesquiterpenoid |
| SO | [3aS-(3aR*,​4R*(Z)​,​5S*,​6E,​10E,​11aS*)]​-2-Butenoic acid, 2-​methyl-​, 2,​3,​3a,​4,​5,​8,​9,​11a-​octahydro-​5-​hydroxy-​6,​10-​dimethyl-​3-​methylene-​2-​oxocyclodeca[b]​furan-​4-​yl ester | sesquiterpenoid |
| SO | Orientalide | sesquiterpenoid |
| SO | [3aS-​(3aR*,​4R*,​5R*,​6E,​10E,​11aS*)​]​-2-​Propenoic acid, 2-​methyl-​, 5-​(acetyloxy)​-​6-​formyl-​2,​3,​3a,​4,​5,​8,​9,​11a-​octahydro-​10-​methyl-​3-​methylene-​2-​oxocyclodeca[b]​furan-​4-​yl ester | sesquiterpenoid |
| SO | [3aS-​(3aR*,​4R*,​5R*,​6E,​10Z,​11aS*)​]​-2-​Propenoic acid, 2-​methyl-​, 10-​[(acetyloxy)​methyl]​-​6-​formyl-​2,​3,​3a,​4,​5,​8,​9,​11a-​octahydro-​5-​methoxy-​3-​methylene-​2-​oxocyclodeca[b]​furan-​4-​yl ester | sesquiterpenoid |
| SP | 2-Propenoic acid, 2-​methyl-​, (3aS,​4S,​5S,​6E,​10Z,​11aR)​-​6-​formyl-​2,​3,​3a,​4,​5,​8,​9,​11a-​octahydro-​5-​hydroxy-​10-​(hydroxymethyl)​-​3-​methylene-​2-​oxocyclodeca[b]​furan-​4-​yl ester | sesquiterpenoid |
| SP | 2-​Propenoic acid,2-methyl-,(3aS,4S,5S,6E,10Z,11aR)-5-ethoxy-6-formyl-2,3,3a,4,5,8,9,11a-octahydro-10-(hydroxymethyl)-3-methylene-2-oxocyclodeca[b]​furan-​4-​yl ester | sesquiterpenoid |
| SO, SP | Pubetallin | sesquiterpenoid |
| SO | 14-Hydroxy-8β-isobutyryloxycostunolide | sesquiterpenoid |
| SO | 9β,14-Dihydroxy-8β-isobutyryloxycostunolide | sesquiterpenoid |
| SO | 8β-Isobutyryloxy-14-al-costunolide | sesquiterpenoid |
| SO | 15-Hydroxy-8β-isobutyryloxy-14-oxo-melampolide | sesquiterpenoid |
| SO | Acanthospermal B | sesquiterpenoid |
| SO | 15-Hydroxy-9α-acetoxy-8β-isobutyryloxy-14-oxo-melampolide | sesquiterpenoid |
| SO | 9α,15-Dihydroxy-8β-isobutyryloxy-14-oxo-melampolide | sesquiterpenoid |
| SO | 9β-Hydroxy-8β-isobutyryloxycostunolide | sesquiterpenoid |
| SO | (1(10)​E,​4E,​8Z)​-​8-​(Angeloyloxy)​-​6α,​15-​dihydroxy-​14-​oxogermacra-​(1(10)​,​4,​8,​11(13)​)​-​tetraen-​12-​oic acid 12,​6-​lactone | sesquiterpenoid |
| SO | (1(10)​E,​4β)​-​8β-​(Angeloyloxy)​-​6α,​14,​15-​trihydroxygermacra-​1(10)​,​11(13)​-​dien-​12-​oic acid 12,​6-​lactone | sesquiterpenoid |
| SG | 2-Methylbut-2-enoic acid,2,3,3a,4,5,8,9,10,11,11a-decahydro-6,10-bis(hydroxymethyl)-3-methylene-2-oxocyclodeca[b]furan-4-yl ester | sesquiterpenoid |
| SG | 2-Propenoic acid, 2-methyl-2,3,3a,4,5,8,9,10,11,11a-decahydro-6,10-bis (hydroxymethyl)-3-methylene-2-oxocyclodeca[b]furan-4-yl ester | sesquiterpenoid |
| SO | Arcotiopicrin | sesquiterpenoid |
| SO | (4β,10E)-6α, 14, 15-Trihydroxy-8β-(isobutyryloxy)germacra-10, 11(13)-diene-12-oic acid 12, 6-lactone | sesquiterpenoid |
| SO | (1(10)​E,​4Z)​-​8β-​(Angeloyloxy)​-​9α,​13-​diethoxy-​6α,​15-​dihydroxy-​14-​oxogermacra-​1(10)​,​4-​dien-​12-​oic acid 12,​6-​lactone | sesquiterpenoid |
| SO | (1(10)​E,​4Z)​-​8β-​(Angeloyloxy)​-​9α-​ethoxy-​6α,​15-​dihydroxy-​13-​methoxy-​14-​oxogermacra-​1(10)​,​4-​dien-​12-​oic acid 12,​6-​lactone | sesquiterpenoid |
| SO | 8β-Isobutyryloxy-1β, 10α-epoxycostunolide | sesquiterpenoid |
| SO | 9β-Hydroxy-8β-isobutyryloxy-1β, 10α-epoxycostunolide | sesquiterpenoid |
| SO | 14-Hydroxy-8β-isobutyryloxy-1β, 10α-epoxycostunolide | sesquiterpenoid |
| SO | 8β, 9β-Dihydroxy-1β, 10α-epoxy-11β, 13-dihydrocostunolide | sesquiterpenoid |
| SP | Germacrene D | sesquiterpenoid |
| SP | Germacra-4(15),5,10(14)-trien-1α-ol | sesquiterpenoid |
| SP | δ-​Cadinene | sesquiterpenoid |
| SP | α-​Cadinol | sesquiterpenoid |
| SP | T-​Muurolol | sesquiterpenoid |
| SP | γ-Muurolene | sesquiterpenoid |
| SP | (1S,4aS,8aR)-1,2,3,4,4a,5,6,8a-Octahydro-7-methyl-4-methylene-1-(1-methylethyl)naphthalene | sesquiterpenoid |
| SP | (1S-cis)-1,2,3,5,6,8a-Hexahydro-4,7-dimethyl-1-(1-methylethyl)naphthalene | sesquiterpenoid |
| SP | α-​Copaene | sesquiterpenoid |
| SP | 1β,6α-Dihydroxy-4(14)-eudesmene | sesquiterpenoid |
| SP | Spathulenol | sesquiterpenoid |
| SP | (+)-4a-Allospathulenol | sesquiterpenoid |
| SO | 11,​12,​13-Trinorguai-​6-​ene-​4β,​10β-​diol | sesquiterpenoid |
| SO | 8β-​(Angeloyloxy)​-​4β,​6α,​15-​trihydroxy-​14-​oxoguaia-​9,​11(13)​-​dien-​12-​oic acid 12,​6-​lactone | sesquiterpenoid |
| SO | 4β,​6α,​15-​Trihydroxy-​8β-​(isobutyryloxy)​-​14-​oxoguaia-​9,​11(13)​-​dien-​12-​oic acid 12,​6-​lactone | sesquiterpenoid |
| SO | [3aR-​(3aα,​4α,​5aβ,​6β,​9aα,​9bβ)​]​-Propanoic acid, 2-​methyl-​, dodecahydro-​6-​hydroxy-​5a-​methyl-​3,​9-​bis(methylene)​-​2-​oxonaphtho[1,​2-​b]​furan-​4-​yl ester | sesquiterpenoid |
| SO | Santamarine | sesquiterpenoid |
| SG | Siegenolide A | sesquiterpenoid |
| SG | Siegenolide B | sesquiterpenoid |
| SP | Carabrone | sesquiterpenoid |
| SP | (3aR,4aS,5S,5aR,6aR)-Octahydro-5-(3-hydroxybutyl)-5a-methyl-3-methylene-2H-cyclopropa[f]benzofuran-2-one | sesquiterpenoid |
| SP | 2,6-Dideacetoxybritanin | sesquiterpenoid |
| SP | α-​Humulene | sesquiterpenoid |
| SO, SP | β-​Caryophyllene | sesquiterpenoid |
| SP | Caryolane-1,9β-diol | sesquiterpenoid |
| SP | Octahydro-​4-​hydroxy-​3α-​methyl-​7-​methylene-​α-​(1-​methylethyl)​-​1H-​indene-​1-​methanol | sesquiterpenoid |
| SP | Opposit-4(15)-en-1β,11-diol | sesquiterpenoid |
| SP | Copaborneol | sesquiterpenoid |
| SP | 2,6-Dimethyl-6-(4-methyl-3-penten-1-yl)bicyclo[3.1.1]hept-2-ene | sesquiterpenoid |
| SP | (6S,7E)-6,9-Dihydroxymegastigma-4,7-dien-3-one | sesquiterpenoid |
| SO | β-​Caryophyllene oxide | sesquiterpenoid |
| SG | Siegesbeckialide I | sesquiterpenoid |
| SG | Siegesbeckialide J | sesquiterpenoid |
| SG | Siegesbeckialide K | sesquiterpenoid |
| SG | Siegesbeckialide L | sesquiterpenoid |
| SG | Siegesbeckialide M | sesquiterpenoid |
| SG | Siegesbeckialide N | sesquiterpenoid |
| SG | Siegesbeckialide O | sesquiterpenoid |
| SG | Glabrescone A | sesquiterpenoid |
| SG | Glabrescone B | sesquiterpenoid |
| SG | Glabrescone C | sesquiterpenoid |
| SO, SP, SG | β-​Sitosterol | steroid |
| SO, SP, SG | β-Daucosterol | steroid glucoside |
| SP | Stigmasterol-​7-​one | steroid |
| SP | stigmast-4-en-one | steroid |
| SP | 5α, 8α-epidioxy-24(R)-methyl-cholesta-6, 22-diene-3β-ol | steroid |
| SP | Stigmasterol | steroid |
| SP | β-​Sitostenone | steroid |
| SP | Peroxyergosterol | Steroid |
| SP | Ursolic acid | Ursane |
| SP | 2β,19α-dihydroxyursolic acid | Ursane |
| SP | 2β-hydroxyursolic acid | Ursane |
| SP | 3-​(Myristoyloxy)​-​2-​(isobutyloxy)​-​4-​methylpentanoic acid | Oxylipin |
| SP | 3-​(Myristoyloxy)​-​2-​hydroxy-​4-​methylpentanoic acid | Oxylipin |
| SP | γ-​Dodecyl-​α-​hydroxy-​γ-​lactone | Oxylipin |
| SG | Sigesbeckin A | Oxylipin |
| SG | Sigesbeckin B | Oxylipin |
| SG | SigesbeckinC | Oxylipin |
| SG | Sigesbeckin D | Oxylipin |
| SG | Sigesbeckin E | Oxylipin |
| SG | Sigesbeckin F | Oxylipin |
| SG | Sigesbeckin G | Oxylipin |
| SO | Siegesoxylipin A | Phyto- Oxylipin |
| SO | Siegesoxylipin B | Phyto- Oxylipin |
| SO | Siegesoxylipin C | Phyto- Oxylipin |
| SO | Siegesoxylipin D | Phyto- Oxylipin |
| SO | Siegesoxylipin E | Phyto- Oxylipin |
| SO | Siegesoxylipin F | Phyto- Oxylipin |
| SO | Siegesoxylipin G | Phyto- Oxylipin |
| SO | Siegesoxylipin H | Phyto- Oxylipin |
| SO | Siegesoxylipin I | Phyto- Oxylipin |
| SO | Siegesoxylipin J | Phyto- Oxylipin |
| SG | lignanoids, Glalignin A | Lignoids |
| SG | lignanoids, Glalignin B | Lignoids |
| SG | lignanoids, Glalignin C | Lignoids |
| SG | lignanoids, Glalignin D | Lignoids |
| SG | lignanoids, Glalignin E | Lignoids |
| SG | Glaneolignin A | Lignoids |
| SG | (+)-isolariciresinol | Lignoids |
| SG | (+)-syringaresinol | Lignoids |
| SG | dihydrodehydrodiconiferyl alcohol | Lignoids |
| SG | tribulusamide A | Lignoids |
| SP | *cis*-​3-​Hexen-​1-​ol |  |
