# Supplementary material for: 44-year journey (1980–2024): scientometric insights into Sigesbeckiae herba and update on its medicinal properties and phytochemicals profile
Source: Chin Med. 2026 Mar 4;21:77. doi: 10.1186/s13020-025-01308-6 (PMC12958691; doi:10.1186/s13020-025-01308-6)
Supplement: Supplementary file 3 [file 13020_2025_1308_MOESM3_ESM.docx]

**Supplementary table 3**: Small organic compounds identified from *Sigesbeckia herba*.

| **Compound Name** | **CAS No.** | **Molecular Formula** | **Species** | **Reference** |
| --- | --- | --- | --- | --- |
| *cis*-3-Hexen-1-ol | 928-96-1 | C6H12O | SP | (Sakuda, 1987) |
| 7,12,14-Hexadecatrien-  10-ynal | 113563-13-6 | C16H22O | SP | (Sakuda, 1987) |
| (*E,E,Z*)-7,12,14-  Hexadecatrien-10-ynal | N/A | C16H22O | SP | (Sakuda, 1987) |
| 7,14-Hexadecadiene-10,  12-diynal | 113563-15-8 | C16H20O | SP | (Sakuda, 1987) |
| 7,12,14-Hexadecatrien-  10-yn-1-ol | 16697-19-1 | C16H24O | SP | (Sakuda, 1987) |
| 9,14,16-Octadecatrien-  12-ynal | 113563-18-1 | C18H26O | SP | (Sakuda, 1987) |
| (*E,Z,Z*)-9,14,16-  Octadecatrien-12-ynal | N/A | C18H26O | SP | (Sakuda, 1987) |
| (*E,E,Z*)-9,14,16-  Octadecatrien-12-ynal | N/A | C18H26O | SP | (Sakuda, 1987) |
| 9,16-Octadecadiene-12,  14-diynal | 113563-20-5 | C18H24O | SP | (Sakuda, 1987) |
| 9,14,16-Octadecatrien-  12-yn-1-ol | 113563-21-6 | C18H28O | SP | (Sakuda, 1987) |
| Acetylene | 74-86-2 | C2H2 | SP | (Sakuda, 1987) |
| Phytol | 7541-49-3 | C20H40O | SO | (Zdero et al.,  1991) |
| Thymohydroquinone dimethyl ether | 14753-08-3 | C12H18O2 | SO | (Zdero et al.,  1991) |
| 19-Acetoxy-12-oxo-  10,11-  dihydrogeranylnerol | 135436-56-5 | C22H36O4 | SO | (Zdero et al.,  1991) |
| 19-Acetoxy-15hydroperoxy-12-oxo13,*14E*-dehydro-10, 11,  14, 15-  tetrahydrogeranylnerol | 135436-57-6 | C22H36O6 | SO | (Zdero et al.,  1991) |
| 19-Acetoxy-15-hydroxy-  12-oxo-13,*14E*-dehydro-  10, 11, 14, 15tetrahydrogeranylnerol | 135436-58-7 | C22H36O5 | SO | (Zdero et al.,  1991) |
| 1-Heneicosanol | 15594-90-8 | C21H44O | SO | (Guo et al.,  1997) |
| Methyl arachidate | 1120-28-1 | C21H42O2 | SO | (Guo et al.,  1997) |
| Ferulic acid | 1135-24-6 | C10H10O4 | SG | (Fu et al.,  1998b) |
| Heptacosanol | 2004-39-9 | C27H56O | SG | (Fu et al.,  1998b) |
| Glycerol monopalmitate | 26657-96-5 | C19H38O4 | SG | (Fu et al.,  1998a) |
| *trans*-Caffeic acid | 501-16-6 | C9H8O4 | SO | (Le et al., 1999) |
| Benzenemethanol | 100-51-6 | C_7_H_8_O | SP | (Gao et al.,  2000) |
| 3,7-Dimethyl-trans-2,6octadien-1-ol | 106-24-1 | C10H18O | SP | (Gao et al.,  2000) |
| 6,10,14-Trimethyl-2pentadecanone | 502-69-2 | C18H36O | SO, SP | (Gao et al.,  2000) |
| (*1E,4E,7E*)-2,5,9,9Tetramethyl-1,4,7cyclodecatriene | 355377-14-9 | C14H22 | SP | (Gao et al.,  2000) |
| 2-Hydroxy-4-isopropyl2,4,6-cycloheptatrien-1one | 499-44-5 | C10H12O2 | SP | (Gao et al.,  2000) |
| (+)-*trans*-*p*-Menth-2-ene | 5256-65-5 | C10H18 | SP | (Gao et al.,  2000) |
| Tetracosanoic acid | 302912-17-0 | C24H48O2 | SP | (Gao et al.,  2003) |
| Tetracosanoic acid, octyl ester | 42233-45-4 | C32H64O2 | SP | (Gao et al.,  2003) |
| Uracil | 66-22-8 | C4H4N2O2 | SP | (Jiang et al.,  2009) |
| Tetracosane carbonic acid | 302912-17-0 | C24H48O2 | SP | (Zhao et al.,  2012) |
| (*E*)-3-(3-Oxobut-1enyl)phenyl dimethylcarbamate | 1415731-47-3 | C13H15NO3 | SP | (Liu et al.,  2012a) |
| 3-(Dodecanoyloxy)-2(isobutyryloxy)-4methylpentanoic acid | 1433993-41-9 | C22H40O6 | SG | (Kim et al.,  2012) |
| *p*-Hydroxy benzaldehyde | 123-08-0 | C7H6O2 | SP | (Wang et al.,  2014b) |
| *m*-Hydroxy-p-methoxy benzaldehyde | 86884-84-6 | C8H8O3 | SP | (Wang et al.,  2014b) |
| 3,4,5-Trimethoxybenzoic acid | 118-41-2 | C10H12O5 | SP | (Wang et al.,  2014b) |
| Monoethyl malonate | 1071-46-1 | C5H8O4 | SP | (Wang et al.,  2014b) |
| *p*-Hydroxylcinnamic acid | 7400-08-0 | C9H8O3 | SP | (Wang et al.,  2014b) |
| 2,6-Di(3-hydroxy-4methoxyphenyl)-3, 7dioxacyclo[3.3.0]octane | 1263045-21-1 | C20H22O6 | SP | (Wang et al.,  2014b) |
| 3-(*m*-Hydroxyl-pmethoxy)-*N*-(2'-*p*hydroxylphenethyl)-*2E*acrylamide | 155174-37-1 | C18H19NO4 | SP | (Wang et al.,  2014b) |
| Succinic acid | 110-15-6 | C4H6O4 | SP, SG | (Fu et al.,  1998b) |
| Dia-aurantiamide acetate | 80780-41-2 | C27H28N2O4 | SP | (Zhao et al.,  2012) |
| Hexadecanoic acid ethyl ester | 628-97-7 | C18H36O2 | SO | (Chang et al.,  2014) |

# References

Chang, C.C., Hsu, H.F., Huang, K.H., Wu, J.M., Kuo, S.M., Ling, X.H., Houng, J.Y., 2014. Anti-proliferative effects of Siegesbeckia orientalis ethanol extract on human endometrial RL-95 cancer cells. Molecules 19(12), 19980-19994.

Fu, H., Cai, S., Feng, R., Lou, Z., 1998a. Chemical constituents of *Siegesbeckia glabrescens* Ⅱ. Chinese Pharmaceutical Journal (Chinese) 33(5), 276-278.

Fu, H., Lou, Z., Cai, S., Hu, X., Zhang, Z., 1998b. Chemical constituents of *Siegesbeckia glabrescens* Ⅰ. Chinese Pharmaceutical Journal (Chinese) 33(3), 140-142.

Gao, H., Li, P., Li, D., Du, X., 2003. Studies on chemical constituents of *Siegesbeckia pubescens* Ⅱ. Chinese Traditional and Herbal Drugs 34(7), 597-599.

Gao, H., Li, P., Wu, W., 2000. Study on volatile oil in stem and leaf of *Siegesbeckia pubescens* Makino. Journal of Norman Bethune University Of Medical Sciences (Chinese) 26(5), 456-457.

Guo, D.a., Zhang, Z., Ye, G., Lou, Z., 1997. Studies on liposoluble constituents from the aerial parts of *Siegesbeckia orientalis* L. Acta Pharmaceutica Sinica 32(4), 282-285.

Jiang, L., Ding, H.-w., Song, S.-j.,2009. Isolation and identification of the chemical constitutents from the whole plant of *Siegesbeckia pubescens* Makino. Journal of Shenyang Pharmaceutical University (Chinese)

6, 6.

Kim, Y.-S., Kim, H., Jung, E., Kim, J.-H., Hwang, W., Kang, E.-J., Lee, S., Ha, B.-J., Lee, J., Park, D., 2012. A novel antibacterial compound from *Siegesbeckia glabrescens*. Molecules 17(11), 12469-12477.

Le, K.N., Nguyen, V.D., Phan, T.S., 1999. Chemistry of *Siegesbeckia orientalis* L., Asteraceae. Hoa Hoc Va Cong Nghiep Hoa Chat 5, 30-32.

Liu, J., Chen, R., Nie, Y., Feng, L., Li, H.-D., Liang, J.-Y., 2012a. A new carbamate with cytotoxic activity from the aerial parts of *Siegesbeckia pubecens*. Chinese Journal of Natural Medicines 10(1), 13-15.

Sakuda, Y., 1987. The constituents of essential oil from *Siegesbeckia pubescens* Makino. Journal of Japan Oil Chemists' Society 36(9), 667-670.

Wang, R., Shi, Y., Wang, Q., Cao, H., 2014b. Chemical constituents from traditional Chinese medicine *Siegesbeckia pubescens*. China Journal of Chinese Materia Medica (Chinese) 39(24), 4811-4815.

Xiang, Y., Fan, C.Q., Yue, J.M., 2005. Novel sesquiterpenoids from *Siegesbeckia orientalis*. Helvetica Chimica

Zdero, C., Bohlmann, F., King, R., Robinson, H., 1991.

Sesquiterpene lactones and other constituents from *Siegesbeckia orientalis* and Guizotia scabra.

Phytochemistry 30(5), 1579-1584.

Zhao, K., Liu, K., Zhao, F., 2012. Active constituents from *Siegesbeckia Pubescens*. Asia-Pacific Traditional Medicine 8(2), 40-42.
